# Genome-Wide Mapping Targets of the Metazoan Chromatin Remodeling Factor NURF Reveals Nucleosome Remodeling at Enhancers, Core Promoters and Gene Insulators

**DOI:** 10.1371/journal.pgen.1005969

**Published:** 2016-04-05

**Authors:** So Yeon Kwon, Valentina Grisan, Boyun Jang, John Herbert, Paul Badenhorst

**Affiliations:** Institute of Biomedical Research, University of Birmingham, Edgbaston, United Kingdom; University of Cambridge, UNITED KINGDOM

## Abstract

NURF is a conserved higher eukaryotic ISWI-containing chromatin remodeling complex that catalyzes ATP-dependent nucleosome sliding. By sliding nucleosomes, NURF is able to alter chromatin dynamics to control transcription and genome organization. Previous biochemical and genetic analysis of the specificity-subunit of *Drosophila* NURF (Nurf301/Enhancer of Bithorax (E(bx)) has defined NURF as a critical regulator of homeotic, heat-shock and steroid-responsive gene transcription. It has been speculated that NURF controls pathway specific transcription by co-operating with sequence-specific transcription factors to remodel chromatin at dedicated enhancers. However, conclusive in vivo demonstration of this is lacking and precise regulatory elements targeted by NURF are poorly defined. To address this, we have generated a comprehensive map of *in vivo* NURF activity, using MNase-sequencing to determine at base pair resolution NURF target nucleosomes, and ChIP-sequencing to define sites of NURF recruitment. Our data show that, besides anticipated roles at enhancers, NURF interacts physically and functionally with the TRF2/DREF basal transcription factor to organize nucleosomes downstream of active promoters. Moreover, we detect NURF remodeling and recruitment at distal insulator sites, where NURF functionally interacts with and co-localizes with DREF and insulator proteins including CP190 to establish nucleosome-depleted domains. This insulator function of NURF is most apparent at subclasses of insulators that mark the boundaries of chromatin domains, where multiple insulator proteins co-associate. By visualizing the complete repertoire of *in vivo* NURF chromatin targets, our data provide new insights into how chromatin remodeling can control genome organization and regulatory interactions.

## Author Summary

In eukaryotes DNA is folded and compacted into manageable units by wrapping around a protein spool of histone proteins to form nucleosomes. By varying the position and dynamics of nucleosomes using energy-dependent chromatin remodeling enzymes, genes can be selectively turned off or on in cells, controlling development and cellular function. Distinct sub-families of ATP-dependent chromatin remodeling enzymes have been characterised. However, their specific nucleosome targets in the genome and how they are recruited to these are not completely defined. Here we have identified nucleosome targets of the conserved higher eukaryotic chromatin remodeling enzyme NURF. Our data indicate three distinct functions for NURF during transcription. NURF organizes nucleosome positions at gene enhancer elements to regulate transcription initiation, but is also required to maintain nucleosome position downstream of the transcription start site of active genes. In addition, we detect NURF remodeling and recruitment at distal insulator sites that are required for functional organisation of the genome. We postulate that NURF function at insulators as well as promoters reflects functional interaction between distant insulators and active promoters, with functional clustering of regulatory elements providing a solution to how chromatin remodeling enzymes engage multiple targets in the genome.

## Introduction

The organization of DNA in nucleosomes has a major function in controlling accessibility of DNA to the protein complexes that process genetic information. By altering nucleosome dynamics, targets for the transcription, replication and repair machineries can be rendered inaccessible or made available. A number of mechanisms exist by which chromatin states can be altered. Post-translational modification of the histone tails (HPTMs) can change associations between histones and DNA, altering chromatin flexibility and conformation (reviewed in Tessarz and Kouzarides [1]). However, these modifications can also act as marks that can be bound by effector complexes that include ATP-dependent chromatin remodeling factors (reviewed in Swygert and Peterson [2]). These multi-subunit protein complexes utilize the energy of ATP hydrolysis to alter nucleosome dynamics. They can be divided into broad families based on the core catalytic subunit and effects on nucleosomes—eviction, sliding or variant histone replacement.

The imitation switch (ISWI) family of ATP-dependent chromatin remodeling factors mediate energy-dependent nucleosome sliding [3, 4]. The nucleosome remodeling factor (NURF) is one of the founding members of this family. Although chromatin remodeling complexes based on ISWI type catalytic subunits are present in all metazoa, NURF is an innovation of the bilateria. NURF complexes are built around a large, bilaterian-conserved, NURF-specific subunit, in *Drosophila* Nurf301/Enhancer of bithorax (E(bx)), in humans BPTF (Bromodomain and PHD finger Transcription Factor) [5, 6]. Like other ISWI-containing complexes NURF catalyzes nucleosome sliding [5, 6], allowing access to transcription factor (TF) binding sites to be regulated and transcription controlled. Consistent with this, mutations in *Nurf301/E(bx)* were initially identified as regulators of the bithorax-complex [7], and subsequently shown to lead to altered transcription regulation of signal cascades including the ecdysone, heat-shock responsive and JAK/STAT pathways [7–9].

Current models for NURF function propose activity at defined enhancers leading to regulation of a restricted set of gene targets. We and others have shown that Nurf301/E(bx) can directly interact with sequence-specific TFs that include the GAGA factor (Trithorax-like (Trl)), ecdysone receptor (EcR), and the repressor Ken [6, 8, 9], suggesting that NURF remodeling is targeted to enhancer elements by direct interactions with TFs. However, transcriptional regulation in metazoa requires complex interplay between regulatory sequences that include not only upstream enhancer elements, but also core promoter regions, and flanking insulators that modulate interactions between enhancers and core promoters. In principle NURF could regulate transcription by remodelling chromatin at any of these elements.

The core promoter regulates transcription initiation by serving as the recognition site for the basal transcription apparatus and determines specificity for upstream enhancers. In bilateria marked diversification of core promoter architecture occurs, with complexes including the canonical TATA-binding protein (TBP) containing complex TFIID, as well as complexes containing TBP-related factors (TRFs) such as the DREF/TRF2 complex, binding distinct core sequences [10–12]. Previous research has indicated that the DREF/TRF2 complex contains the ISWI, Caf1 and Nurf-38 subunits of NURF. However, involvement of the full NURF complex in DREF/TRF2 function is unclear. While peptides corresponding to the Nurf301/E(bx) subunit were not mapped to the DREF/TRF2 complex [13], mutants lacking the Putzig subunit of DREF/TRF2 phenocopy *Nurf301E(bx)* mutants [14], suggesting NURF interacts with DREF/TRF2 and may influence core promoter function.

In turn, intra- and inter-chromosomal communication between the core promoter and enhancer regions can be controlled by insulator elements. These were first defined in *Drosophila* based on enhancer blocking function which can be mediated by combinations of three DNA-binding proteins—Boundary Element Associated Factor (BEAF), CCCTC-Binding Factor (CTCF) and Suppressor of Hairy wing (Su(Hw))–and two associated proteins, Centrosomal Protein 190 (CP190) and Modifier of mdg4 (Mod(mdg4)) [15].

To provide an unbiased assessment of regulatory elements at which NURF acts, we have used whole genome MNase-sequencing to map at base pair resolution nucleosomes that require NURF for positioning. In parallel, we have used chromatin immunoprecipitation sequencing (ChIP-Seq) of the Nurf301/E(bx) specificity subunit to identify sites of stable recruitment of NURF. To our knowledge this is the first comprehensive base pair resolution map of *in vivo* nucleosome targets of a metazoan chromatin remodeling enzyme. Our data indicate NURF action at three distinct transcription regulatory elements. In addition to upstream enhancer elements, we identify a novel function of NURF in orchestrating nucleosome spacing downstream of the +1 nucleosome on active genes regulated by DREF/TRF2. Moreover, we also detect NURF remodeling and recruitment at distal insulator sites, where NURF interacts with DREF and known insulator proteins including CP190 to establish nucleosome-depleted domains.

## Results

### Nucleosome mapping identifies NURF target nucleosomes

To identify sites of NURF activity in the genome we profiled nucleosome distribution in *Drosophila* WT and NURF deficient primary macrophages (hemocytes). As a first step normalized tag densities of uniquely-mapped reads were determined in a sliding 50 bp window across the genome and log2 fold changes between mutant and wild-type (WT) were used to define regions of altered read densities, we term shifts. Using this approach, we identified 47,000 shifted nucleosomes in Nurf301/E(bx) deficient larval hemocytes. Assuming an averaged female *Drosophila* genome size of 175 Mb [16], this corresponded to <5% of nucleosomes, indicating that NURF remodelling was deployed locally at discrete nucleosomes and not globally over large arrays of nucleosomes. Local action by NURF was confirmed by visualizing NURF nucleosome shifts over entire chromosome arms using Hilbert plots (S1 Fig), which indicated NURF nucleosome shifts were randomly distributed across both the X-chromosome and autosomes with no evidence of large domains of remodeling.

**Fig 1:**
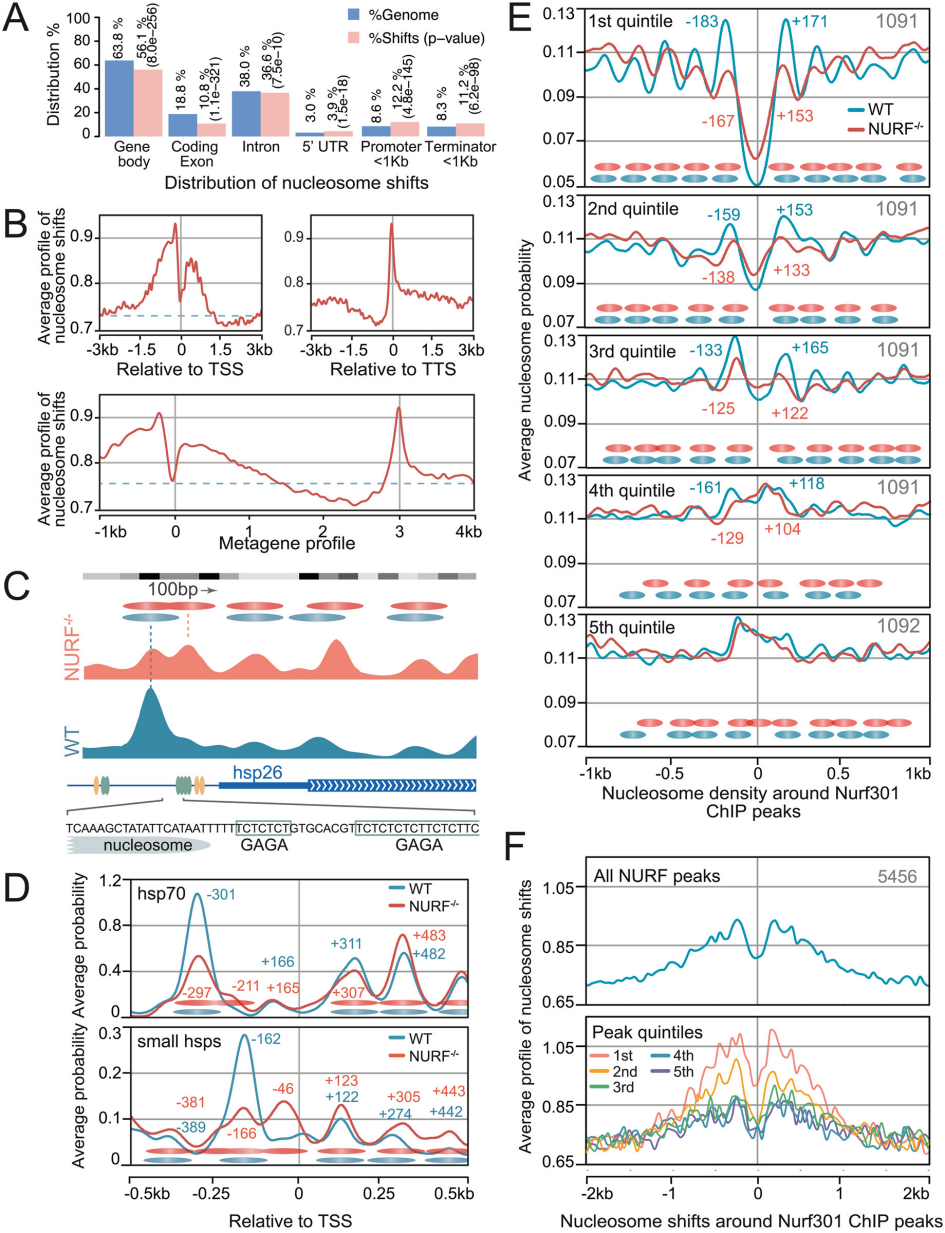
Genome-wide mapping of NURF target nucleosomes. (A) Distribution of nucleosome shifts relative to gene features compared with percentage of the genome occupied by features. (B) Averaged profile of nucleosome shifts relative to TSSs and TTSs reveals NURF-dependent nucleosome shifts at upstream regulatory elements, the +1 nucleosome and TTSs. Metagene profile shows averaged distribution of nucleosome shifts over entire gene bodies, with gene bodies scaled to 3 kb.(C) Nucleosome probability at *hsp26* a known NURF target. A nucleosome is positioned between GAGA elements (green ovals) in wild type (WT) cells but not maintained in *Nurf301/E(bx)* mutants. Yellow ovals indicate HSF-binding sites. (D) Averaged nucleosome probability traces at *hsp70* (*hsp70Aa*, *hsp70Ab*, *hsp70Bb*, *hsp70Ba*, *hsp70Bbb* and *hsp70Bc*) and small heat shock (*hsp22*, *hsp23*, *hsp26* and *hsp27*) loci confirm promoter nucleosome redistribution in *Nurf301/E(bx)* mutants. (E) NURF sites were determined by Nurf301/E(bx) ChIP-Seq, and peaks ordered according to strength of associated ChIP signal to identify 5456 robust sites, which were further divided into quintiles from highest (1st) to lowest (5th) signal. Averaged nucleosome probability flanking these sites was determined in wild type (WT) and *Nurf301/ E(bx)* (NURF^-/-^) samples revealing extensive changes in nucleosome organisation flanking NURF sites. (F) These trends are confirmed by plotting averaged nucleosome shifts relative to peak co-ordinates for all NURF sites, or NURF sites classified into quintiles based on Nurf301/E(bx) ChIP signal.

The distribution of NURF nucleosome shifts relative to known gene features was then determined. Consistent with the hypothesis that NURF regulates transcription by remodelling enhancer nucleosomes, we observed significant enrichment of nucleosome shifts on 5’ regulatory elements and under-representation on coding exons and gene bodies (Fig 1A, S2 Fig). However, NURF nucleosome shifts were also enriched on 5’UTRs and 3’ regulatory elements suggesting additional targets of NURF. To investigate these in greater detail, we determined the averaged distribution of NURF nucleosome shifts relative to all transcription start sites (TSSs) and transcription termination sites (TTSs) (Fig 1B). This defined distinct functions for NURF in transcription regulation. Firstly, a broad enrichment of nucleosome shifts was detected extending 1.5 kb upstream of the transcription start site, consistent with remodelling at enhancers. Secondly, NURF-dependent remodelling was also observed downstream of the TSS, with a peak detected at the +1 nucleosome position and shifts detected above background for approximately 1.2 kb into the gene body, corresponding to 5–6 nucleosomes. Finally, NURF was also required to maintain nucleosome organisation at transcription terminators.

These activities were defined at base pair resolution by mapping individual nucleosome dyad positions, which were then used to generate a continuous nucleosome density estimation that describes the probability of a nucleosome at each base pair in the genome. By generating nucleosome density estimates for both WT and *Nurf301/E(bx)* mutant backgrounds, nucleosomes that require NURF to maintain position could be discriminated. The validity of this mapping procedure was confirmed by analysing nucleosome positions on heat-shock loci, the targets originally used to identify NURF [17]. Nucleosome positions on heat-shock promoters are well documented, with the TF Trl binding GAGA sequences to recruit NURF, which in turn remodels nucleosomes establishing a nucleosome-depleted region [6, 17]. Consistent with this, WT hemocyte nucleosome probabilities showed a clearly positioned nucleosome adjacent to Trl-binding GAGA elements (Fig 1C). However, in *Nurf301/E(bx)* mutants this nucleosome position was not maintained and new nucleosome positions were detected (Fig 1C and 1D).

We next confirmed that changes in nucleosome position in *Nurf301/E(bx)* mutants corresponded to sites of NURF localisation determined by ChIP-seq. Nurf301/E(bx) ChIP peaks were ordered according to strength of associated signal, and sub-divided into quintiles from highest (1st) to lowest (5th) Nurf301/E(bx) signal. Analysis of nucleosome position flanking these sites demonstrated consistent changes in nucleosome position in *Nurf301/E(bx)* mutants (Fig 1E). This was confirmed by peaks in nucleosome shifts flanking NURF ChIP peaks (Fig 1F). In addition, microarrays were used to examine expression of other chromatin regulators in *Nurf301/E(bx)* mutant hemocytes. Analysis of expression of 667 genes that have assigned GO (gene ontology) molecular functions related to chromatin and transcription (S3A Fig) showed no substantial decreases in expression in other chromatin modifying and remodelling complexes (S3B Fig). Taken together, these data suggest that the nucleosome shifts and changes in nucleosome distribution determined above were direct measures of NURF activity.

### NURF activity at predicted TF binding sites

**Fig 2:**
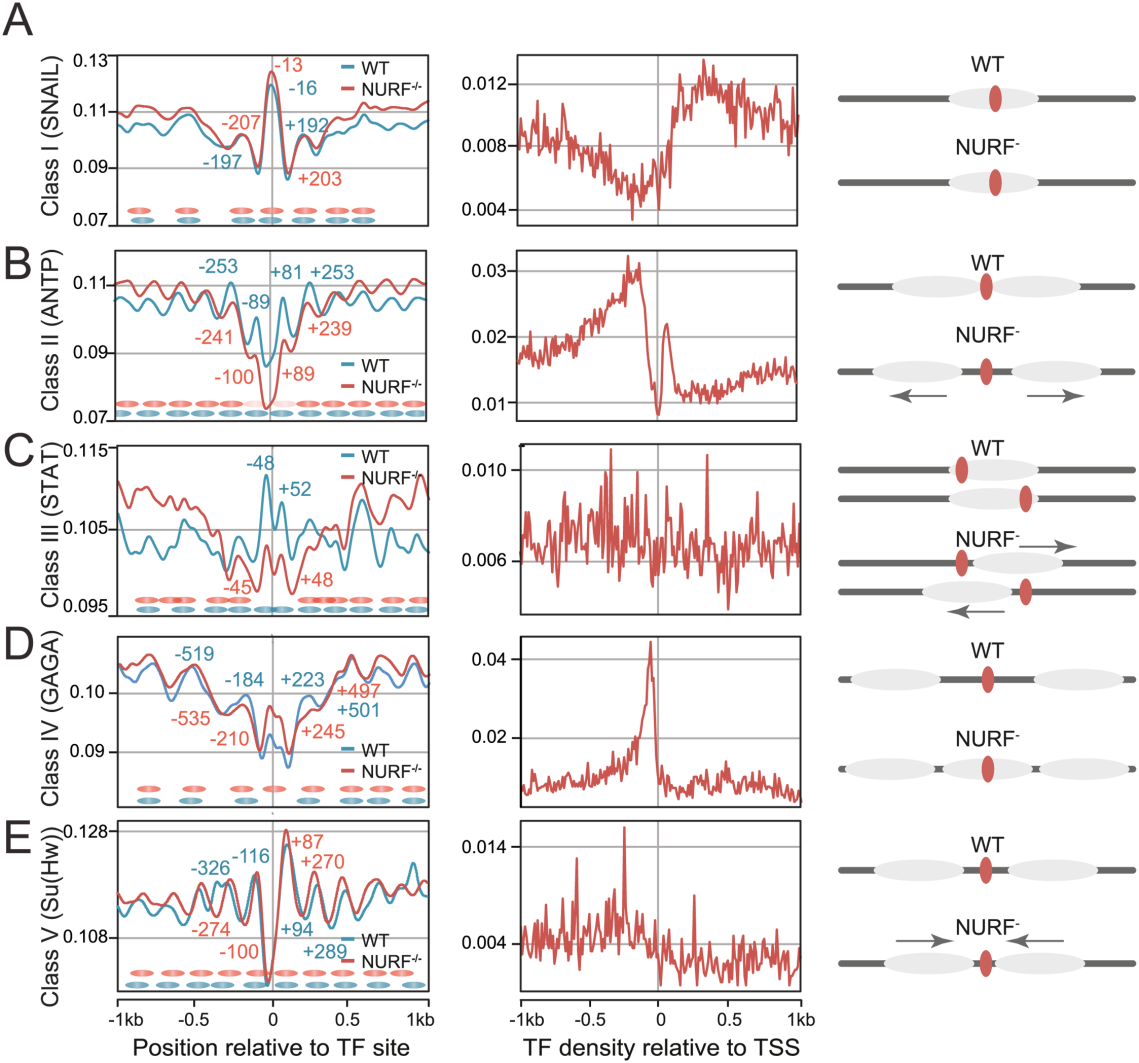
Nucleosome organization around predicted transcription factor binding sites. Predicted binding sites for *Drosophila* TFs were determined using MEME and averaged nucleosome probability plots flanking these sites generated for both wild type (WT) and *Nurf301/E(bx)* mutants. Averaged profile plots of predicted TF-binding sites relative to the TSS were also generated. Five categories of nucleosome organization around predicted TF sites were distinguished: (A) NURF-independent positions, (B) NURF-dependent nucleosomes adjacent to TF sites, (C) NURF-dependent nucleosomes with TF sites in the nucleosome entry/exit points, (D) NURF-dependent nucleosome-depleted domains that flank TF sites, and (E) barrier function where NURF-dependent nucleosome-depleted domains are flanked by regular nucleosome arrays.

NURF nucleosome remodelling flanking Trl-binding sites, and the broad peak of nucleosome shifts over the 5’ upstream regions of genes, were consistent with NURF’s proposed function of collaborating with TFs at enhancers to slide nucleosomes and control transcription. To establish if this was a specific property of Trl, or whether NURF cooperates with other families of TFs to remodel enhancer nucleosomes, we screened *Drosophila* TFs with known DNA-binding consensi for NURF-dependent nucleosome positioning and remodeling activity. Using DNA-binding consensi, predicted genome-wide binding sites for individual TFs were computed and averaged nucleosome density flanking these in WT and NURF-deficient hemocytes determined (Fig 2, S4 and S5 Figs). Based on flanking nucleosome organization and changes in NURF mutants, TFs could be assigned to one of five categories.

Predicted binding sites for class I TFs were independent of NURF and located within nucleosomes, (Fig 2A). Binding sites for the remaining classes of TFs (Class II-V), were flanked by NURF-dependent nucleosome positions and revealed three distinct modes by which NURF and TFs could potentially influence enhancer nucleosome position. Thus, class II and class III predicted TF-binding sites were located either immediately adjacent to a nucleosome (Fig 2B), or at nucleosome entry/exit points (Fig 2C), respectively. In *Nurf301/E(bx)* mutants these positions were lost and chromatin flanking the site was more accessible, suggesting these TFs potentially could tether NURF to mediate directional nucleosome sliding. In contrast, class IV predicted TF-binding sites, exemplified by Trl (Fig 2D), were located in extended nucleosome-depleted regions, which show higher nucleosome signals in *Nurf301/E(bx)* mutants. The final mode was observed flanking consensi for DNA-binding proteins including Su(Hw) (Fig 2E). Binding sites for this class of TF were localized in a nucleosome-depleted domain flanked by organized nucleosome arrays that migrate towards the predicted binding sites in *Nurf301/E(bx)* mutants.

As controls, analysis of averaged nucleosome position flanking TF sites that were clustered or isolated was broadly consistent with trends observed with all sites (S6 Fig). In addition, microarray analysis of third instar larval hemocytes showed no change in TF expression in *Nurf301/E(bx)* mutants (S3C Fig), excluding indirect effects of changes in expression in these factors on nucleosome organisation.

### NURF-dependent nucleosome shifts on active genes

**Fig 3:**
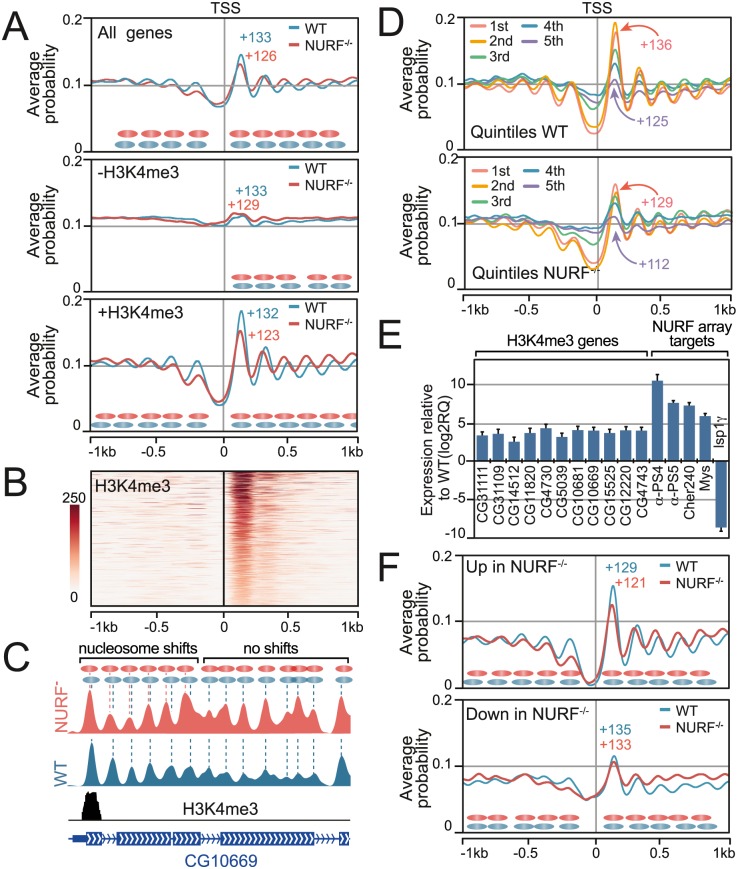
NURF maintains nucleosome spacing on active genes. (A) Averaged nucleosome probability relative to all TSSs, and active (+H3K4me3) or inactive (-H3K4me3) TSSs. +1 nucleosome dyad position is indicated. (B) Heatmap of H3K4me3 in hemocytes. (C) Nucleosome probability trace at *CG10699* in wild type (WT) and *Nurf301/E(bx)* mutant hemocytes corroborates NURF-dependent nucleosome positioning of six nucleosomes downstream of the TSS. (D) Genes were binned according to expression quintile from highest (1st) to lowest (5th) and averaged nucleosome probability relative to the TSS determined. (E) Transcript levels of genes containing H3K4me3 were increased in *Nurf301/E(bx)* mutants. Levels determined by real-time PCR relative to WT normalized using *rp49*. (F) Shifts in +1 nucleosome dyad position (labeled) only occur for genes with increased expression in *Nurf301/E(bx)* mutants.

However, NURF function was not restricted to remodelling nucleosomes at enhancers. Our initial comparative analysis of nucleosome tag densities detected potential nucleosome shifts not only upstream but also downstream of the TSS (Fig 1B). To elucidate the basis of this, we used the calculated continuous nucleosome density estimations to compare nucleosome position downstream of all TSSs in the *Drosophila* genome in both WT and *Nurf301/E(bx)* mutant backgrounds. This showed the expected nucleosome distribution in WT samples with a nucleosome-depleted region flanking the TSS, a well-positioned +1 nucleosome and a regular downstream array of nucleosomes (Fig 3A). In *Nurf301/E(bx)* mutants, a superficially similar distribution was observed, with a regular array of nucleosomes downstream of the TSS. However absolute nucleosome position was shifted towards the TSS for the first six nucleosomes following the TSS (+1 to +6 nucleosome positions).

This analysis included both active and inactive TSSs. To discriminate if NURF differentially affected transcriptionally active versus inactive promoters, we categorized active and inactive TSSs in hemocytes by profiling the hemocyte distribution of the H3K4me3 HPTM, which decorates the +1 nucleosome of active genes (Fig 3B). Profiling averaged nucleosome probability relative to active (+H3K4me3) and inactive (-H3K4me3) TSSs showed that clearly defined nucleosome arrays were only detected downstream of active TSSs and not inactive TSSs (Fig 3A). On active genes in WT cells the +1 nucleosome was located at +132 bp relative to the TSS, but was shifted closer to the TSS at +123 bp in *Nurf301/E(bx)* mutants (Fig 3A, +H3K4me3). Nucleosome shifts towards the TSS in *Nurf301/E(bx)* mutants were propagated over the next five nucleosomes after which the nucleosome array reset and limited differences between mutant and WT arrays were observed.

These trends using averaged nucleosome density profiles were confirmed on randomly selected, individual H3K4me3-containing genes. For example, nucleosome positions detected on the *CG10699* gene showed a 10 bp change in position of the +1 nucleosome in *Nurf301/E(bx)* mutants (Fig 3C), and shifts expanding over successive nucleosomes, but limited to the first six nucleosomes. This restriction of nucleosome shifts to the first six nucleosomes downstream of the TSS, agreed well with our initial comparative analysis of nucleosome tag-density, where we only detected nucleosome shifts within the first 1.2 kb downstream of the TSS (Fig 1B).

Calculating average nucleosome repeat length downstream of the TSS on active and inactive genes in both genetic backgrounds showed that nucleosome repeat length on active genes decreased from 175 bp in WT samples to 170 bp in *Nurf301/E(bx)* mutants. In contrast, on inactive genes, the extrapolated WT average nucleosome repeat length of 185 bp was unchanged in *Nurf301/E(bx)* mutants. Similar analysis of nucleosome positions at other genomic regions, for example flanking exon-intron boundaries, also showed no change in nucleosome spacing in *Nurf301/E(bx)* mutants. Taken together we conclude that NURF has a specific nucleosome positioning and spacing function downstream of active TSSs.

### Loss of NURF increases expression of active genes

To establish if this NURF nucleosome spacing function depends on the absolute level of transcription, normalized WT and *Nurf301/E(bx)* mutant hemocyte gene expression levels were determined by Affymetrix microarray profiling. TSSs were binned into quintiles based on normalized WT transcript level from high (1st) to low (5th), and averaged nucleosome position flanking TSSs of each quintile determined in both backgrounds. We observed that, while the WT +1 nucleosome dyad location was shifted downstream as expression level increased (from +125 bp in the lowest expression quintile to +136 bp in the highest quintile), for all quintiles +1 nucleosome positions were relocated towards the TSS in *Nurf301/E(bx)* mutants (Fig 3D). The extent of this movement was constant for all quintiles indicating that NURF-dependent nucleosome spacing on active genes is independent of absolute transcript level.

We next examined the consequences of this spacing change on expression of active genes. Our initial assumption was that active gene expression would be reduced in *Nurf301/E(bx)* mutants. Surprisingly, real-time RT-PCR (Fig 3E) showed increased expression of active (+H3K4me3) genes in *Nurf301/E(bx)* mutant hemocytes. Furthermore, using whole genome expression profiles to categorize TSSs with increased or decreased expression in *Nurf301/E(bx)* mutants, and profiling averaged nucleosome densities flanking the corresponding TSSs, indicated that only TSSs up-regulated in *Nurf301/E(bx)* mutants demonstrated signature NURF-dependent +1 nucleosome shifts (Fig 3F). No changes in +1 nucleosome position were observed on TSSs with decreased expression (Fig 3F). We conclude that NURF dampens expression of transcriptionally active genes.

### NURF targets DREF-responsive active promoters

**Fig 4:**
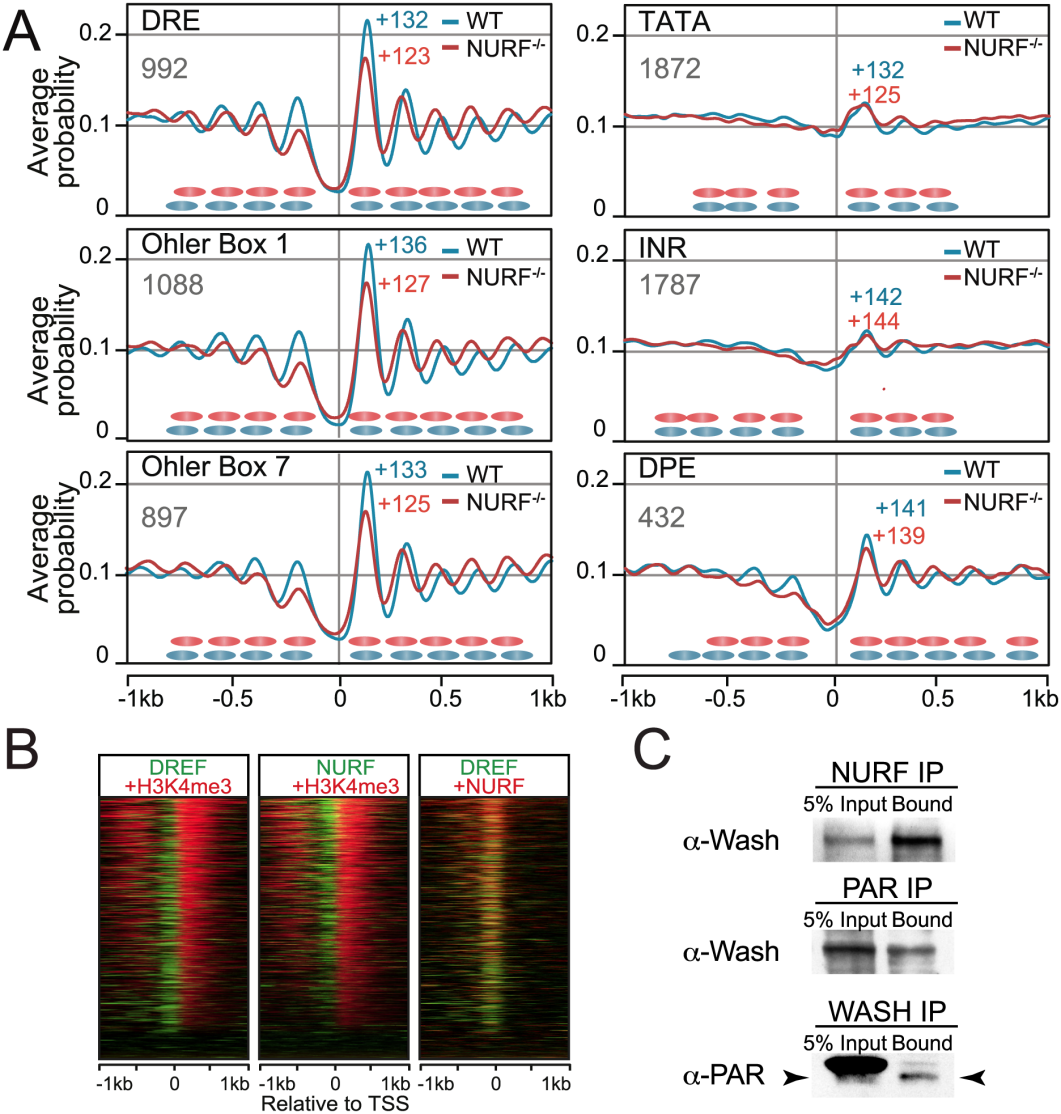
NURF remodeling and core promoter architecture. (A) TSSs were categorized based on associated core promoter motifs and averaged nucleosome probability plots flanking the TSS of each category generated for both wild type (WT) and *Nurf301/E(bx)* mutants. +1 nucleosome dyad position is labeled. +1 nucleosome shifts were detected at promoters containing DREs and Ohler box 1 and 7 consensi (TRF2/DREF targets). (B) Heatmaps of NURF, DREF and H3K4me3 signals relative to the TSS for genes ordered according to the H3K4me3 signal strength. Colocalization of NURF (green) and DREF (red) signals is indicated by yellow merge. (C) NURF immunoprecipitated from S2 soluble nuclear fraction associates with the DREF/TRF2 subunit Wash. Reciprocal co-immunoprecipitation from S2 soluble nuclear fraction using anti-Wash and anti-PAR antibodies indicates that Wash is PARylated. An abundant PARylated protein partially overlaps with Wash in Input but is not immunoprecipitated by anti-Wash antibodies. Wash is indicated by arrowheads. 5% Input is loaded as control in all experiments.

We demonstrated a specific NURF nucleosome spacing function downstream of active TSSs. To discriminate if NURF promoter targeting was mediated by core promoter components we classified TSSs based on associated core promoter elements. Nucleosome organisation flanking these distinct categories of TSSs was then determined in WT and *Nurf301/E(bx)* mutants. This showed clear NURF activity on promoters containing DREs and Ohler motifs 1, 5, and 7 (Fig 4A, S7 Fig), elements enriched on TRF2-bound promoters [18] and house-keeping core-promoters [10]. This promoter class exhibited a well-defined +1 nucleosome and robust downstream nucleosome array that was shifted towards the TSS in *Nurf301/E(bx)* mutants. NURF activity on DREF-dependent promoters was confirmed by comparison of Nurf301/E(bx) and published DREF ChIP-Seq profiles [19]. Heatmaps indicated that DREF and Nurf301/E(bx) were enriched and colocalized at active TSSs (Fig 4B), confirmed by co-immunoprecipitation which showed physical association of NURF and the DREF/TRF2 complex subunit Washout (Wash) in S2 cell extracts (Fig 4C).

In contrast, promoters containing TATA, INR, MTE and DPE motifs exhibited less well-defined nucleosomal arrays (Fig 4A, S7 Fig). On INR-, MTE- and DPE-containing promoters the +1 nucleosome was located approximately 10 bp further into the gene body (+142 bp), a location characteristic of paused promoters [20] (S8 Fig). Significantly, no relative change in position of these nucleosomes was observed in *Nurf301/E(bx)* mutants (Fig 4A, S7 Fig). Taken together our data indicate predominant NURF function on active promoters that are targets of DREF/TRF2.

### NURF controls nucleosome organization at insulator elements

**Fig 5:**
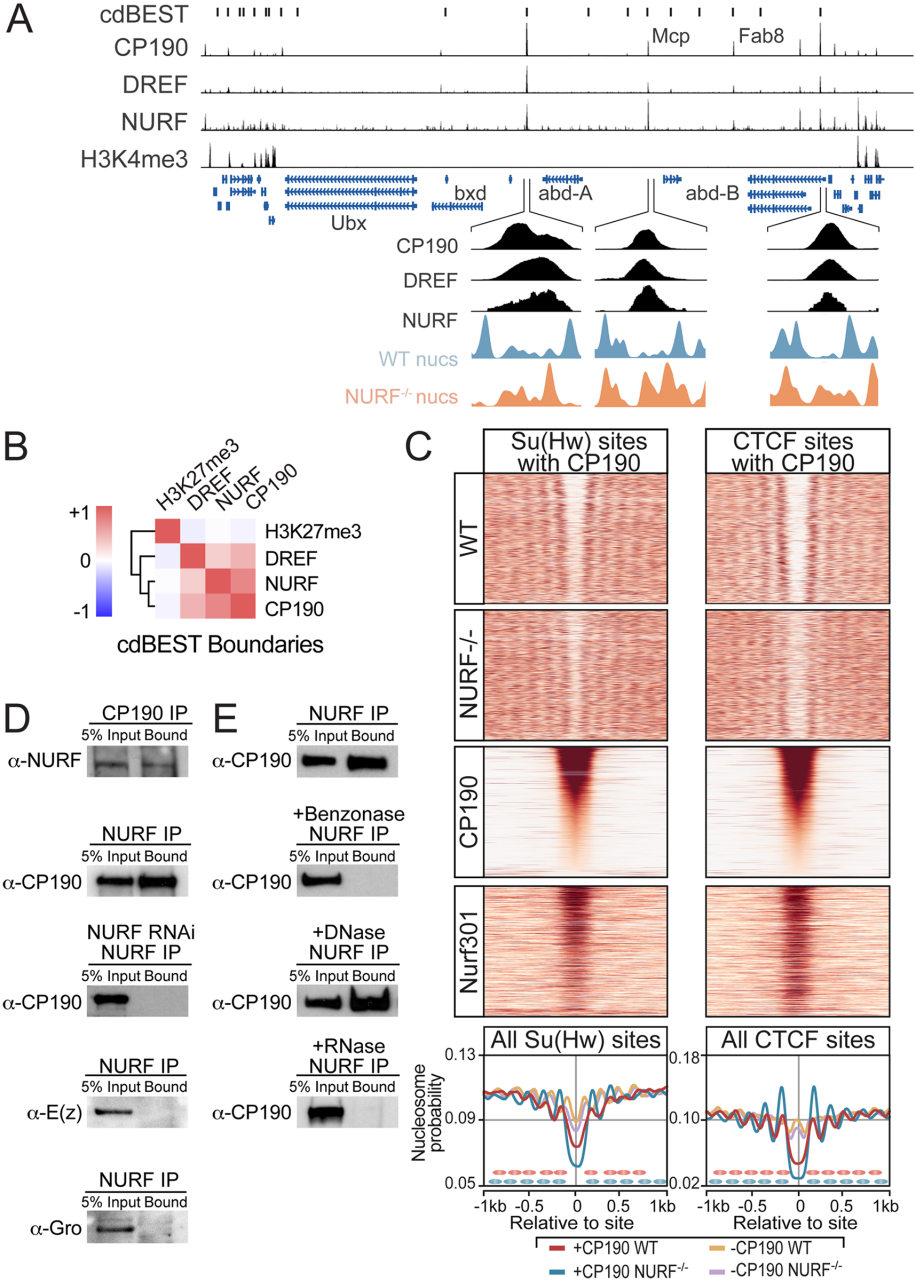
NURF organizes nucleosomes around insulator sites. (A) Genome browser view showing NURF, CP190, DREF and H3K4me3 distribution at the bithorax complex. Predicted (cdBEST) [21] and known insulator sites (Mcp and Fab8) are indicated. (B) NURF, CP190 and DREF signals are well correlated at predicted insulator sites (cdBEST). (C) Heatmap of nucleosomes and Nurf301/E(bx) at Su(Hw) and CTCF sites that also contain CP190, ordered according to CP190 signal (threshold of at least 50 reads at peak co-ordinate). Averaged nucleosome probability at all Su(Hw) and CTCF sites that either contain (+CP190) or lack (-CP190) CP190 reveals that insulator nucleosome-depleted domain requires associated CP190. (D) Reciprocal co-immunoprecipitation from S2 soluble nuclear fraction using anti-Nurf301 and anti-CP190 antibodies indicates proteins physically associate. As negative controls, anti-Enhancer of zeste (E(z)) and anti-Groucho (Gro) antibodies do not immunoprecipitate CP190, and anti-Nurf301 pull-down from *Nurf301*-RNAi knockdown cells fails to immunoprecipitate CP190. (E) Anti-Nurf301 antibodies immunoprecipitate CP190 from MNase-treated soluble chromatin. This association is lost following Benzonase or RNase A treatment. 5% Input is loaded as control in all experiments.

In addition to targeting active promoters, inspection of Nurf301/E(bx) ChIP-Seq profiles revealed NURF localisation to isolated elements that corresponded to previously defined insulator elements, including the Fab8 and Mcp boundaries in the bithorax complex (Fig 5A). This concurred with our analysis of nucleosome distribution flanking TF-binding sites (Fig 2), which showed Su(Hw) and BEAF insulator proteins collaborate with NURF to establish nucleosome-depleted domains. NURF targeting to insulators was verified by ChIP-Seq of the core insulator component CP190, which showed good correlation with Nurf301/E(bx) at predicted insulator sites genome-wide (Fig 5B, cdBEST Boundaries [21]). Inspection of boundaries in the bithorax complex demonstrated that CP190 and Nurf301/E(bx) co-localized in nucleosome-depleted regions in WT hemocytes, at which nucleosomes were detected in *Nurf301/E(bx)* mutants (Fig 5A, expanded view).

Nucleosome profiling flanking ChIP peaks for the DNA-binding insulator components CTCF, Su(Hw) and BEAF [22] showed that these occurred within nucleosome-depleted domains flanked by ordered nucleosome arrays. However, levels of the associated cofactor CP190 determined nucleosome reorganization at these sites. Thus clear nucleosome reorganization was observed when insulator-binding sites for CTCF (Fig 5C, S9 Fig), Su(Hw) (Fig 5C, S10 Fig), BEAF (S11 Fig), and Mod(mdg4) (S12 Fig) were ordered according to CP190 level. Segregation of binding sites for Su(Hw), and BEAF into those with or without CP190 confirmed that CP190 determined nucleosome organization around insulator elements. In *Nurf301/E(bx)* mutants, nucleosome-depleted domains at CP190-containing insulator sites were reduced in size and nucleosomes repositioned. Consistent with the discriminating role of CP190, reciprocal co-immunoprecipitation revealed that NURF and CP190 were physically associated in S2 cell extracts (Fig 5D and 5E).

### DREF/TRF2 colocalizes with NURF at insulators

**Fig 6:**
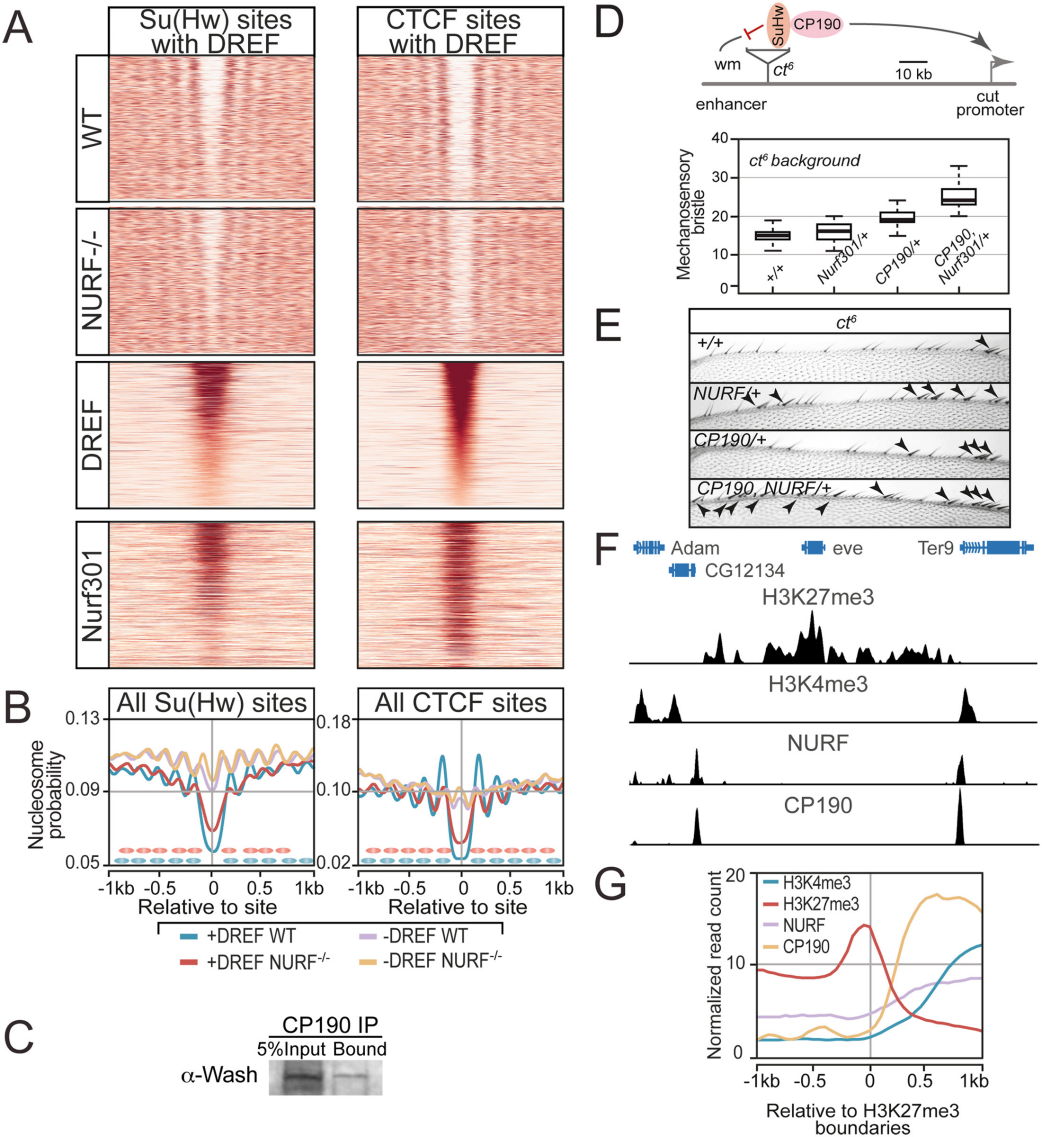
NURF nucleosome remodeling at insulator sites requires DREF. (A) Heatmap of nucleosomes and Nurf301/E(bx) at Su(Hw) and CTCF sites that also contain DREF ordered according to DREF signal. (B) Averaged nucleosome probability at all Su(Hw) and CTCF sites that either contain (+DREF) or lack (-DREF) DREF reveals that insulator nucleosome-depleted domain requires associated DREF (threshold of 50 reads at peak co-ordinate). (C) Anti-CP190 antibodies immunoprecipitate Wash. 5% Input is loaded as control in all experiments. (D) The enhancer blocking action of the *ct*^*6*^ gyspy transposon, reduces mechanosensory bristle number on the anterior wing margin. Simultaneous removal of one copy of Nurf301 and CP190 partially restores mechanosensory bristles. Box-plots indicate at least 100 independent determinations. (E) Anterior wing margin showing mechanosensory bristles (arrowheads). (F) The H3K27me3-containing *eve* domain is flanked by NURF and CP190 peaks that separate it from surrounding H3K4me3-containing active genes. (G) Genome-wide profile of NURF, CP190, H3K4me3 and H3K27me3 signals at the boundaries of H3K27me3-enriched domains reveals NURF and CP190 flank H3K27me3 domains.

Taken together, our data show NURF can localize to both core promoters and insulators. Our initial working assumption was that these distributions result from separate targeting of NURF either to promoters by DREF/TRF2 or to insulators by CP190. Surprisingly, inspection of DREF ChIP profiles indicated colocalization with Nurf301/E(bx) and CP190 at insulator elements in the bithorax complex (Fig 5A) and predicted insulator sites genome-wide (Fig 5B). Moreover, as we had observed for CP190 (Fig 5C), levels of DREF determined nucleosome reorganization at these insulator sites. Thus, nucleosome profiling flanking Su(Hw) and CTCF binding sites showed that clear nucleosome-depleted domains were only detected when insulator-binding sites contained DREF (Fig 6A). Those lacking DREF were occupied by nucleosomes (Fig 6B). In *Nurf301/E(bx)* mutants, nucleosome-depleted domains at DREF-containing insulator sites were reduced in size. Consistent with DREF action at insulators, co-immunoprecipitation showed that the DREF/TRF2 subunit Wash and CP190 were physically associated in S2 cell extracts (Fig 6C). In addition, Wash was poly ADP ribose (PAR) modified (Fig 4C), a hallmark of destabilized chromatin [23] that occurs at insulator sites.

Functional requirement for NURF at an insulator *in vivo* was confirmed using the known enhancer blocking function of *gypsy* retrotransposons. These contain binding sites for Su(Hw), which can recruit insulator components to establish a functional insulator. When integrated between the wing enhancer and promoter of the *cut* gene in the *ct*^*6*^ mutation, *gypsy* elements suppress *ct* wing expression and disrupt the adult wing margin. This normally is decorated with approximately 90 mechanosensory bristles. Loss of bristles in *ct*^*6*^ mutants can be used as a sensitive assay for *gypsy* insulator function. In the absence of other mutations *ct*^*6*^ alleles reduce mechanosensory bristle number to 14 (Fig 6D and 6E). Loss of one copy of either Nurf301/E(bx) or CP190 increases mechanosensory bristle number, while simultaneous removal of one copy of both shows synergistic increase in bristle number, demonstrating functional cooperation between NURF and insulator proteins at the *gypsy* insulator.

Our data demonstrated that insulators acted on by NURF are targeted by multiple proteins including DREF and CP190. Such co-association of multiple insulator proteins is a feature of subclasses of insulator elements that often mark the boundaries of H3K27me3 domains or topologically associating domains (TADs) [24, 25], suggesting these are the targets of NURF. Evidence for this was provided by the *Drosophila even-skipped* locus. In S2 cells this is spanned by a well-defined H3K27me3 domain, flanked by active H3K4me3 containing genes with peaks containing both NURF and CP190 detected at the boundaries between these domains (Fig 6F). Consistent with NURF function at such boundaries, we observed negative correlation genome-wide between H3K27me3 and either NURF, CP190, or DREF (Fig 5B), and inverse correlation between NURF, CP190, or DREF and H3K27me3 levels at H3K27me3 domain boundaries (Fig 6G).

### NURF collaborates with insulator proteins at core promoters

**Fig 7:**
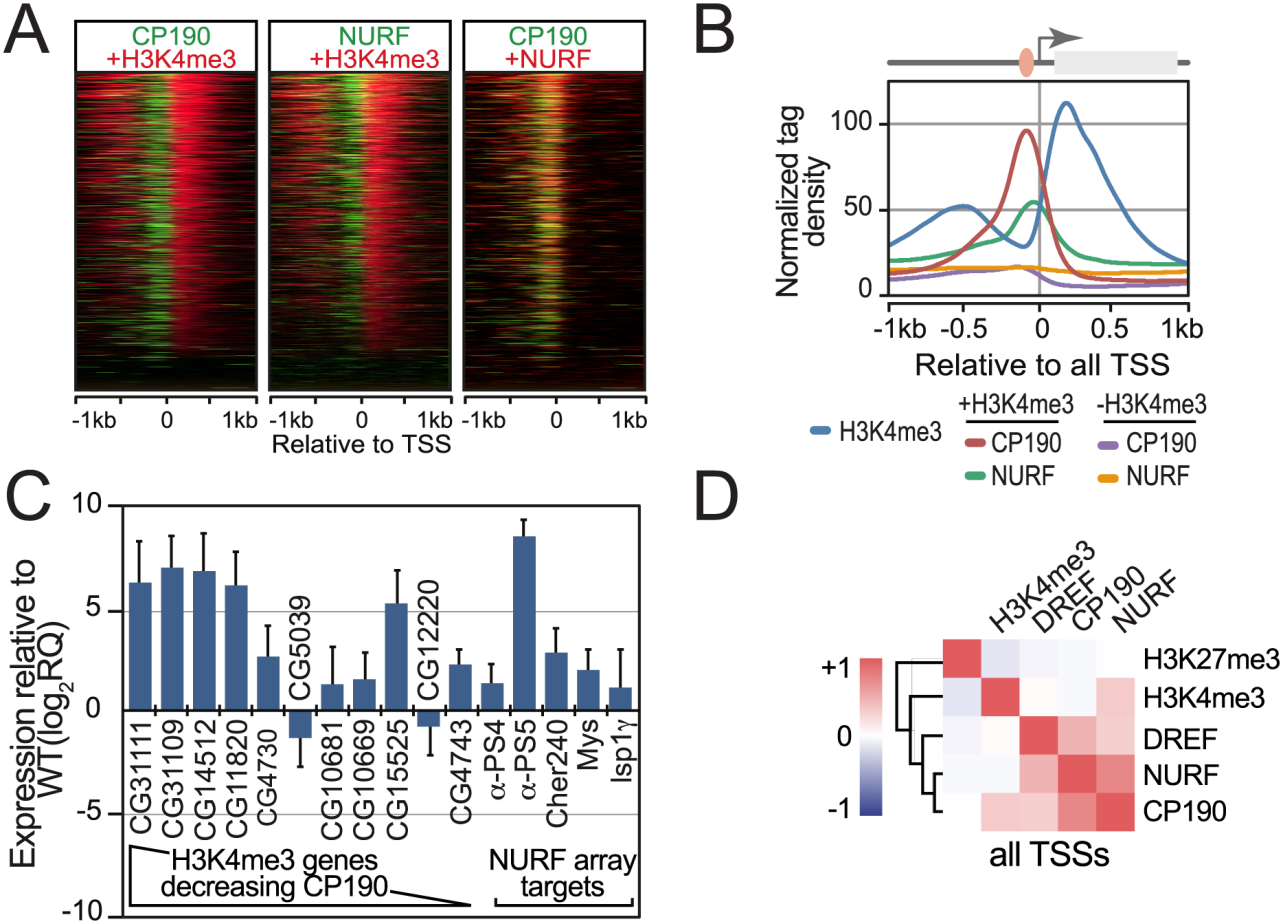
NURF interacts with insulator components at promoters. (A) Heatmaps of NURF, CP190 and H3K4me3 signals relative to the TSS for genes ordered according to the H3K4me3 signal strength. Colocalization of NURF (red) and CP190 (green) signals is indicated by yellow merge. (B) Normalized NURF and CP190 ChIP tag density relative to TSSs. TSSs are categorized as active (+H3K4me3) or inactive (-H3K4me3) based on H3K4me3 signal. (C) Transcript levels of NURF targets in CP190 mutant hemocytes, determined by real-time PCR relative to WT and normalized to *rp49*. Transcripts are ordered according to associated H3K4me3. (D) NURF, CP190 and DREF signals are well correlated at TSSs.

Finally, as Nurf301/E(bx), DREF and CP190 colocalize at insulators, and Nurf301/E(bx) and DREF also overlap at active promoters, we tested whether insulator components may also be similarly localized to active promoters. Comparison of CP190 and Nurf301/E(bx) ChIP-Seq profiles in S2 cells reveals that CP190 was present at TSSs. However, CP190 was only detected on active genes (Fig 7B, compare +H3K4me3 and –H3K4me3 CP190 traces). This was confirmed by heatmaps of CP190 and NURF signals relative to H3K4me3 around TSSs, which show colocalization of signals on active promoters (Fig 7A and 7D).

CP190 binding to both active promoters and distant insulator elements resembles the reported association of TAF3 with CTCF that mediates looping between distal enhancer elements and the proximal promoter [26], raising the possibility that NURF, DREF and CP190 co-localization to insulators and proximal promoters reflects functional interaction between these elements. To test whether increased expression of active genes observed in NURF mutants (Fig 3E) was due to impaired insulator function, we tested whether loss of CP190 similarly increased active gene expression. However, although expression of some genes increased in CP190 mutants, up-regulation was not consistently observed for all genes (Fig 7C), suggesting that NURF plays additional roles distinct from insulator function at these targets.

## Discussion

Modulation of nucleosome dynamics by ATP-dependent chromatin remodelling enzymes has the potential to regulate all chromatin templated reactions. Key to understanding functions in diverse processes ranging from transcription to repair and replication is to elucidate *in vivo* targets and mechanisms by which ATP-dependent chromatin remodelling activity is deployed. Here we have used mononucleosome mapping of WT and *Nurf301/E(bx)* mutant cells to discriminate nucleosomes remodelled by NURF *in vivo*. We detect interactions with transcription factors and insulator components that direct NURF activity to both gene promoters and distant regulatory elements. To our knowledge this is the first comprehensive base pair resolution map of *in vivo* nucleosome targets of a metazoan chromatin remodeling enzyme. Our data indicate NURF remodelling occurs at discrete sites in the genome, but we speculate that by mediating local chromatin reorganization NURF can profoundly impact genome organization and long-range regulatory interactions.

A central question in the ATP-dependent chromatin remodelling field is whether remodelers are highly abundant, affecting all nucleosomes and genome function generally, or more restricted factors that are recruited to discrete sites to regulate specific nuclear processes. Early estimates of *Drosophila* ISWI abundance [17], and disruption of entire polytene X chromosomes in *Nurf301/E(bx)* mutants [7, 27], were consistent with global function of NURF. However, highly-specific changes in gene expression observed in *Nurf301/E(bx)* mutants suggest more localized effects on nucleosome positioning [7–9]. Data generated here confirm local and not global nucleosome disruption by NURF. Fewer than 5% of nucleosomes genome-wide are remodelled by NURF and large domains of nucleosome reorganisation were not detected by Hilbert curve analysis. Our data in diploid primary hemocytes is fully consistent with array-based analysis of nucleosomes at selected genome regions in ISWI mutant polytene salivary glands which fail to show global nucleosome disruption [28].

NURF targets defined here include upstream enhancers, consistent with the original isolation of NURF as a TF cofactor and initial transcriptome analysis [7–9, 17]. Our data show NURF collaborates with TFs at enhancers to remodel nucleosomes organisation around predicted TF binding sites. We distinguish several modes of NURF-dependent nucleosome organisation at predicted TF sites: precise positioning of nucleosomes adjacent to TF sites; disruption of nucleosomes surrounding TF sites; and a barrier function in which TF sites establish a nucleosome-depleted domain and organize flanking nucleosome position. In combination these data point to distinct modalities by which TFs may deploy NURF to achieve alternate functional outcomes. A caveat, however, is that these data were generated using predicted TF-binding sites based on binding consensi. While available ChIP-Seq datasets of actual Su(Hw), BEAF and Trl binding sites confirm these trends, it remains formally possible that some predicted sites may not be occupied by TFs and/or that TF-binding may be lost in *Nurf301/E(bx)* mutants. Nevertheless, these results are broadly consistent with *in vitro* experiments demonstrating for example how GAL4 provides a “barrier” that competes with nucleosomes for occupancy at DNA targets [29, 30] or, alternatively, how tethering of remodelers like CHD1 can mediate directional nucleosome sliding [31].

However, our data reveal an additional nucleosome spacing function for NURF downstream of active TSSs including house-keeping targets of TRF2/DREF. The consequences of this are distinct from remodelling at enhancers, which mediates high-level changes in transcription of specific developmental/signal transduction pathways, instead exerting a general dampening effect on active gene transcription. Our data indicate that NURF shifts nucleosomes in the 3’direction away from the TSS, similar to yeast ISW1b and CHD1 remodelers [32] and also increases spacing between nucleosomes. This spacing may facilitate recruitment of factors with nucleosome-spacing dependent binding, like the Rpd3(S) complex [33, 34]. In yeast, Rpd3(S) represses intragenic transcription from cryptic initiation sites [35]. One consequence of decreased spacing in NURF-deficient cells may be the failure to recruit repressors of cryptic initiation and increase in spurious transcription, consistent with our observed up-regulation of active gene expression in *Nurf301/E(bx)* mutants.

NURF localisation and activity has parallels with yeast ISWI remodelling complexes, which show recruitment to TSSs but action on gene bodies [32, 36]. We detect binding of NURF to nucleosome depleted regions (NFRs) upstream of the TSS, consistent with yeast ISWI localization, where NFRs provide extended linkers required for remodelling [36]. In our case, this localization is reinforced by interactions with basal transcription factors. In particular, NURF colocalizes and interacts physically and functionally with the TBP-related TRF2/DREF complex. It has been postulated that diversification of core promoter factors and evolution of TRF2 has driven the transcriptional complexity that facilitated the evolution of the bilaterian body plan [37]. It is intriguing that this is accompanied by the emergence of bilaterian-specific chromatin remodeling enzymes. Unlike the SWI/SNF2 and INO80/SWR1 complexes, which show conservation of most subunits throughout eukaryotic lineages, ISWI-complexes like NURF exhibit distinct non-catalytic subunits in bilateria. We speculate that core promoter diversification demands co-evolution of new remodeler complexes to accommodate increasing regulatory complexity.

Promoter localization of NURF is likely further stabilized by NURF binding HPTMs that decorate the +1 nucleosome [38, 39] but, also may be mediated by direct DNA binding. Nurf301/E(bx) contains two N-terminal AT-hook domains which bind AT-rich DNA, and have been shown to be required for full NURF nucleosome sliding activity and nucleosome-binding *in vitro* [6]. A precedent for this is provided by studies of the related SWR1 remodelling complex where targeting to promoter elements is mediated by the DNA-binding SWC2 subunit [40]. It has been suggested recently that remodeler peaks observed at active TSSs may be “phantom” artefacts of the ChIP procedure and should be treated with caution [41]. In our case, however, we observe nucleosome remodelling flanking NURF ChIP signals and NURF binds HPTMs that decorate the +1 nucleosome that flanks the ChIP sites [38, 39].

It is formally possible that some changes in nucleosome positioning observed in *Nurf301/E(bx)* mutants are not the consequence of loss of NURF ATP-dependent nucleosome-sliding activity, but rather that NURF acts stoichiometrically and non-catalytically at some sites, binding and physically occupying linker DNA in a manner incompatible with nucleosome formation. Changes in nucleosome position could thus potentially be due to the lack of NURF physical presence at some sites. The ability of remodelers to engage in either “traditional” catalytic ATP-dependent nucleosome sliding versus and non-catalytic modes would allow diversification of remodeler function, and offer the potential that allosteric modulation (by for example histone modifications) could switch complexes between different modes of remodeling to program distinct local chromatin architectures. The use of strains containing catalytically inactive NURF induced by expressing mutant forms ISWI, in which ATPase activity is eliminated but complex assembly unaffected, could offer one approach to experimentally investigate this.

It is also important to consider that these experiments were performed in cells in which function of other remodeling complexes was unaffected. Systematic profiling of yeast chromatin remodeler distributions indicates overlapping domains of function. As such it is possible that NURF is functionally redundant with other chromatin remodeling enzymes [42]. Thus, targets and nucleosome reorganization identified here may under-represent complete NURF function. There may be sites at which NURF function can be substituted by other chromatin remodeling enzymes in *Nurf301/E(bx)* mutants. Indeed initial analysis of yeast remodeling enzymes required depletion of three remodeling enzymes (*isw1*, *isw2* and *chd1*) to observe substantial effects on nucleosome organisation [43]

Finally, our data reveal that NURF interacts with the insulator elements and interacts with components including CP190 and DREF. Activity of NURF at insulators is consistent with our initial studies showing that *Nurf301/E(bx)* mutants modify *bithorax* mutations and that *Nurf301* corresponds to *Enhancer of bithorax E(bx)* [7]. The *bithorax* mutations are caused by *gypsy* transposons that bind Su(Hw) and act as ectopic insulators to disrupt expression. NURF remodeling at insulator elements is also consistent with results showing that NURF is required for *Drosophila* cell-based insulator/enhancer blocking assays [44]. This appears to be a conserved function as studies in vertebrates show that NURF mediates chromatin barrier function at the chicken β-globin locus [45] and interacts with CTCF to regulate gene expression in mammals [46].

Interestingly, colocalization between Nurf301/E(bx) and insulator proteins is not only detected at distal insulator regions but also at active promoters. We postulate that overlap of insulator components and NURF at insulators as well as promoters reflects functional interaction between distant insulators and active promoters as has been speculated for CTCF at mammalian promoters [26, 47]. It is tempting to speculate that functional clustering of regulatory elements provides a solution to how chromatin remodeling enzymes engage targets in the genome. Three dimensional clustering of targets in proximity would allow rapid recapture of ATP-dependent chromatin remodelers at distinct regulatory elements that require nucleosome reorganisation.

## Materials and Methods

### Genetics and *Drosophila* strains

*Nurf301*^*2*^, *Cp190*^*1*^ and *Cp190*^*2*^ alleles were as described [7, 48]. The *Nurf301*^*2*^ allele is an EMS-induced mutation that encodes a glutamine to stop codon substitution (aa 545) that truncates Nurf301 after the first PHD finger and which behaves genetically as a null allele. Unless stated flies were raised at 25°C.

### Hemocyte isolation and fixation

Third instar larvae were sexed and primary hemocytes collected from wild-type and *Nurf301*^*2*^ mutant female third instar larvae as described previously [9]. Briefly larvae were ripped in batches of 50 third instar larvae into HyQ-CCM3 insect medium (Thermo Fisher Scientific) containing protease inhibitors (Complete, Roche). Cells were fixed with 1% formaldehyde in 1XPBS for 15 minutes at 25°C and pelleted at 400*g* for five minutes. Cells were washed three times with ice cold 1XPBS containing protease inhibitors and stored as pellets at -80°C until required.

### Cell culture

S2-DRSC cells were cultured at 25°C in Insect-XPRESS medium (Lonza) containing 10% FCS. S2 cells for standard ChIP were fixed with 1% formaldehyde and washed as described above.

### Micrococcal nuclease (MNase) digestion

Hemocyte preparations from 1000 larvae were thawed, pooled and resuspended in buffer A (15 mM Tris (pH 7.4), 15 mM NaCl, 60 mM KCl, 0.34 M sucrose, 1 mM DTT, 25 mM sodium metabisulfite, 0.5 mM spermidine, 0.15 mM spermine). Cells were homogenized with a pellet pestle. CaCl_2_ was added to a final concentration of 1 mM, 800U MNase (Worthington) added, and the sample incubated for 12 minutes at 16°C to liberate mononucleosomes. Digestion was stopped by adding an equal volume of Stop buffer (0.1 M Tris (pH 8.5), 0.1 M NaCl, 50 mM EDTA, 1% SDS) and samples centrifuged at 17,400*g* for 10 minutes to purify soluble chromatin. Formaldehyde cross-links were removed by addition of NaCl to 150 mM, one-tenth volume Proteinase K (Roche) and incubation at 65°C overnight. DNA was purified by phenol/chloroform extraction and ethanol precipitation, treated with RNase (Promega) at 37°C for 1 hour and then run on 2.2% recovery FlashGels (Lonza). Mononucleosomal DNA was pipetted from recovery wells in FlashGel Recovery Buffer and DNA subjected to further round of phenol/chloroform extraction and ethanol precipitation. Libraries were generated from two biological replicates for each genotype, sequenced, and reads pooled for nucleosome mapping.

For MNase-ChIP experiments, hemocytes from 1000 larvae were processed as above but soluble chromatin after Stop buffer addition was diluted in ChIP dilution buffer (16.7 mM Tris (pH 8.1), 167 mM NaCl, 1.2 mM EDTA, 0.1% SDS, 1.1% Triton X-100), and processed for ChIP as described below.

### Chromatin immunoprecipitation (ChIP) and sequencing library preparation

ChIP was performed as described in [38] with the following modifications. Samples were pre-cleared using Protein G-conjugated Dynabeads (Invitrogen) for 30 minutes at room temperature, followed by incubation with antibody coated Protein G-conjugated Dynabeads (Invitrogen) for 2.5 hours at room temperature. Immune complexes were recovered by magnetic selection, and washed once with low salt buffer (20 mM Tris (pH 8.1), 150 mM NaCl, 2 mM EDTA, 0.1% SDS, 1% Triton X-100), once with High salt buffer (20 mM Tris (pH 8.1), 500 mM NaCl, 2 mM EDTA, 0.1% SDS, 1% Triton X-100), once with LiCl immune complex wash buffer (10 mM Tris (pH 8.1), 1 mM EDTA, 0.25 M LiCl, 1% IGEPAL CA-630, 1% deoxycholic acid) and twice with TE buffer for five minutes each at room temperature. ChIP DNA was eluted using two washes of elution buffer (1% SDS, 0.1 M NaHCO_3_) for 15 minutes at room temperature. Cross-links were reversed as described above and ChIP DNA purified using 1.8 volumes Agencourt AMPure XP beads (Beckman Coulter). The following antibodies were used: rabbit anti-NURF301, rabbit anti-H3K4me3 (17–614, Millipore), rabbit anti-CP190 [49]. DNA for MNase-Seq and ChIP-Seq was end-repaired and sequencing libraries prepared using a SOLiD Fragment Library Construction Kit (Life Technologies). ChIP DNA was barcoded using the SOLiD Fragment Library Barcoding Kit Module 1–16. Sequencing libraries were run on a SOLiD 4 genome analyzer.

### Nucleosome mapping and ChIP-peak detection

SOLiD reads for nucleosome mapping were mapped to the *Drosophila* genome (BDGP R5/dm3 Assembly) in color space using Bowtie [50] and filtered for high quality reads. Nucleosome density profiles were generated using F-seq [51]. To determine regions with altered nucleosome position between wild-type and mutant cells chromosomes were divided into 50 bp windows and read number in each bin determined. Bins with log2 fold change greater than 2 between mutant and wild-type were identified as regions with nucleosome shifts. ChIP reads were mapped to the *Drosophila* genome (BDGP R5/dm3 Assembly) using the bioscope mapping tool (Life Technologies). Reads were then filtered for high quality reads where read quality was greater than 15 using Samtools [52]. ChIP peaks were called using MACS [53] and MACS ChIP wiggle tracks were then imported into Galaxy and filtered to select peak signals. Tools to generate Pearson correlation coefficients for ChIP-Seq profiles at defined genomic regions, averaged signal density relative to defined genomic regions and heatmaps of nucleosome and ChIP-Seq signals at defined regions were generated using the Cistrome package [54]. Publically available data tracks GSM762836, GSM762837, GSM762838, GSM762839, GSM762840, GSM762841, GSM762842, GSM762843, GSM762844, GSM762845, GSM762846, GSM762847, GSM762848, GSM762849 [25] were used to define developmentally-stable insulator peaks. Nucleosome sequence files have been deposited at European Nucleotide Archive (Study Accession PRJEB12941).

### Whole genome expression analysis

*w*^*1118*^ and *Nurf301*^*2*^ wandering third instar larvae were staged using the blue-gut method and hemocytes isolated in batches of 50 larvae as described previously [9, 38]. mRNA was isolated from hemocytes isolated from the equivalent of 1000 animals using Trizol as described [9, 38] and mRNA amplified and labeled using the GeneChIP Eukaryotic Small Sample Target Labeling Assay VII (Affymetrix). Triplicate labeled mRNA samples were hybridized to GeneChip Drosophila Genome Arrays (Affymetrix). Statistical analysis was carried out using R version 3.1.2 (http://www.R-project.org) and the *gcrma* and *limma* libraries of Bioconductor version 3.0 (http://www.bioconductor.org). Expression values were computed using *gcrma* [55]. Array datasets are available through ArrayExpress (accession number E-MTAB-4537).

### Immunoprecipitation (IP) experiments

Soluble nuclear fraction (SNF) was prepared from *Drosophila* S2 cells using a modification of the protocol of Wysocka and colleagues [56] to prepare nuclei. Nuclear pellets were extracted using extraction buffer (10mM HEPES (pH 7.9), 400 mM KCl, 3mM MgCl_2_, 5% Glycerol, 0.5 mM DTT, 1 mM Sodium Metabisulphite, 1x Protease inhibitors (Complete, Roche)) for 1 hour on ice with gentle swirling. Extracts were clarified by centrifugation at 100,000 *g* for 1 hour at 4°C. SNF was dialysed against extraction buffer adjusted to 100mM KCl. Protein G-conjugated Dynabeads (Invitrogen) were blocked by incubation in blocking buffer (1xPBS, 5mg/ml BSA, 1x Protease inhibitors (Complete, Roche)) and antibodies bound by incubation overnight at 4°C. SNF was diluted into bind buffer (1XPBS, 5mg/ml BSA, 0.1% Tween-20, 1XProtease inhibitors) incubated with antibody-coated Protein G-conjugated Dynabeads for 2.5 hrs with rotation at 4°C. Beads were washed four times, 10 minutes each, using wash buffer (1xPBS, 0.1% Tween-20). Bound proteins were eluted by boiling in 1XSDS-PAGE sample buffer. 5% input run as a loading control for all IPs.

Additional IPs were performed using soluble chromatin prepared according to the protocol of Wysocka and colleagues [56]. Nuclei were lysed and chromatin (predominantly mononucleosomal) liberated by digestion with MNase (Worthington). Soluble chromatin was diluted into binding buffer and incubated with antibody-coated Protein G-conjugated Dynabeads and washed as above. For RNase A, Benzonase and DNase I treatments, beads were pelleted and incubated in digestion buffer (1XPBS, 2.5mM MgCl_2_, 0.5mM CaCl_2_ 0.1% Tween-20) containing the respective enzymes at 37°C for 10 minutes, followed by three further washes in wash buffer. Bound proteins were eluted as described above.

Antibodies used were rabbit anti-NURF301, rabbit anti-CP190 [49], mouse anti-Gro (anti-Gro was deposited to the DSHB by C. Delidakis), mouse anti-E(z) (sc-25903, Santa Cruz Biotechnology), mouse anti-Wash (P3H3-Wash was deposited to the DSHB by S. Parkhurst) and mouse anti-pADPr (Mab 10H, sc-56198, Santa Cruz Biotechnology).

### Real-time RT-PCR analysis

For confirmation of microarray expression data, mRNA was isolated from wild-type and mutant hemocytes using μMacs columns according to manufacturer’s instructions (Miltenyi Biotec, Auburn, CA) and reverse transcribed by Superscript II (Invitrogen) at 42°C. Primer sets used are listed in S1 Table.

## Supporting Information

S1 Fig Visualizing NURF-dependent nucleosome shifts on whole chromosome arms using Hilbert plots. (A) Schematic illustrating a Hilbert plot. These are continuous space-filling curves in which one-dimensional data (in this case location of nucleosome shifts along a chromosome arm) is mapped in two dimensions allowing visualization of all data simultaneously. In this procedure a unit square is progressively divided into smaller quadrants and the one-dimensional line corresponding to each chromosome arm folded such that it passes through the center of each quadrant. The iterative folding has the advantage of allowing all data to points to be observed simultaneously and preserves locality, points that co-localize on the line typically co-map on the curve. In the example two features are plotted—in red (closely juxtaposed arrays of peaks), and in green peaks that are well separated from their neighbours. When the entire chromosome arm is viewed as in a genome browser window, it is impossible to discriminate between these two features due to the low resolution of the whole chromosome arm view. However, if these features are plotted as a Hilbert plot, as the linear arm is folded through smaller and smaller quadrants resolution increases and it becomes possible to discriminate closely juxtaposed and well separated features. In this context, extended domains of nucleosome re-organization would result in clusters of shifts being detected as extended clusters/domains on the Hilbert plot. (B) Hilbert plots of NURF-dependent nucleosome shifts on all chromosome arms.
(TIF)

S2 Fig Distribution of NURF target nucleosomes. (A) Distribution of nucleosome shifts relative to chromosome arms compared with genome average of features. (B) Promoter distribution of nucleosome shifts compared with genome average of features. (C) Gene body distribution of nucleosome shifts compared with genome average of features. (D) Distribution of nucleosome shifts downstream of terminators compared with genome average of features.
(TIF)

S3 Fig Expression of transcription factors and putative chromatin regulators in *Nurf301* mutant hemocytes. (A) To distinguish whether *Nurf301* mutation affects expression of transcription factors or chromatin regulators that could indirectly affect nucleosome organisation in *Nurf301* mutant hemocytes GO Molecular Function assignments (Flybase) were used to identify 667 targets of interest. Venn diagram indicates respective sub-categories of molecular function. (B) Expression of this set of 667 genes was analysed in *Nurf301* mutant hemocytes relative to control wild-type hemocytes. Eight genes display elevated expression in *Nurf301* mutant hemocytes, twelve show reduced expression. Of these, nine are male-expressed genes and, as nucleosome profiles were analysed in female hemocytes, these could be excluded from the analysis. None of the remaining eleven genes have known functions in control of global chromatin organisation or nucleosome positioning suggesting that the effects of *Nurf301* mutants on nucleosome organisation are unlikely to be mediated through indirect effects on expression of other factors. (C) No change in expression of transcription factors previously analysed for effects on nucleosome organisation was observed. Expression of selected genes previously identified to be up-regulated (up-regulated controls) or down-regulated (down-regulated controls) in *Nurf301* mutant hemocytes are displayed as controls. Change in expression is listed as log fold change in *Nurf301* mutant hemocytes relative to wild-type hemocytes.
(TIF)

S4 Fig Nucleosome organization around predicted transcription factor binding sites. (A) Predicted binding sites for *Drosophila* TFs Asense, BEAF, Biniou, Broad, Brinker, Caudal, Crocodile, Deformed, Engrailed, Ets were determined using MEME and averaged nucleosome probability plots flanking predicted TF sites generated for both wild type (WT) and *Nurf301* mutant populations. Five categories of nucleosome organization around TF sites could be distinguished. Dyad position of the +1 nucleosome is labeled. (B) Averaged profile plots of predicted TF-binding sites relative to the TSS.
(TIF)

S5 Fig Nucleosome organization around predicted transcription factor binding sites. (A) Predicted binding sites for *Drosophila* TFs Ftz, Hr46, Mef2, Repo, Runt, Scute and Zerknult were determined using MEME and averaged nucleosome probability plots flanking predicted TF sites generated for both wild type (WT) and *Nurf301* mutant populations. Five categories of nucleosome organization around TF sites could be distinguished. Dyad position of the +1 nucleosome is labeled. (B) Averaged profile plots of predicted TF-binding sites relative to the TSS.
(TIF)

S6 Fig Nucleosome organisation surrounding clustered or isolated TF-binding sites. To discriminate whether nucleosome profiles were affected by clustering of TF binding sites, sites for (A) Snail, (B) Antp, (C) Stat and (D) Su(Hw) were divided into those with at least 2 sites within 50 bp (clustered sites) or those that did not possess neighbouring sites (isolated). Averaged nucleosome probability plots flanking these sites generated for both wild type (WT) and *Nurf301/E(bx)* mutants.
(TIF)

S7 Fig NURF remodeling and core promoter architecture. (A) TSSs were categorized based on core promoter motifs associated with developmentally regulated transcripts, TATA-box, initiator (INR) element, TCT motif, Motif Ten Element (MTE) and the downstream core promoter element (DPE). Averaged nucleosome probability plots flanking the TSS of each category generated for both wild type (WT) and *Nurf301* mutants. (B) TSSs were categorized based on core promoter motifs associated with DREF/TRF2 targets, the DRE and Ohler Boxes 1,5,6,7,8. Averaged nucleosome probability plots flanking the TSS of each category generated for both wild type (WT) and *Nurf301* mutants. Dyad position of the +1 nucleosome is labeled.
(TIF)

S8 Fig Nucleosome position at paused and elongating promoters. TSSs were categorized as stalled or active (not stalled). Averaged nucleosome probability plots flanking the TSS were generated for both wild type (WT) and *Nurf301* mutants at (A) all TSSs, and TSSs with (B) paused polymerase or (C) active not stalled polymerase. Dyad position of the +1 nucleosome is labeled.
(TIF)

S9 Fig Nucleosome reorganization at CTCF sites. (A) Heatmap of nucleosomes in wild-type and *Nurf301* mutant hemocytes at all CTCF sites ordered according to CTCF signal. CP190 signal is shown for comparison. (B) Heatmap of nucleosomes in wild-type and *Nurf301* mutant hemocytes at CTCF sites that contain CP190 ordered according to CP190 signal. CP190 and CTCF signal is shown for comparison. (C) Heatmap of nucleosomes in wild-type and *Nurf301* mutant hemocytes at CTCF sites that lack CP190 ordered according to CTCF signal. CTCF signal is shown for comparison. Graph shows averaged nucleosome probability at CTCF sites that either contain (+CP190) or lack (-CP190) in wild-type and *Nurf301* mutant backgrounds.
(TIF)

S10 Fig Nucleosome reorganization at Su(Hw) sites. (A) Heatmap of nucleosomes in wild-type and *Nurf301* mutant hemocytes at all Su(Hw) sites ordered according to Su(Hw) signal. CP190 signal is shown for comparison. (B) Heatmap of nucleosomes in wild-type and *Nurf301* mutant hemocytes at Su(Hw) sites that contain CP190 ordered according to CP190 signal. CP190 and Su(Hw) signal is shown for comparison. (C) Heatmap of nucleosomes in wild-type and *Nurf301* mutant hemocytes at Su(Hw) sites that lack CP190 ordered according to Su(Hw) signal. Su(Hw) signal is shown for comparison. Graph shows averaged nucleosome probability at Su(Hw) sites that either contain (+CP190) or lack (-CP190) in wild-type and *Nurf301* mutant backgrounds.
(TIF)

S11 Fig Nucleosome reorganization at BEAF sites. (A) Heatmap of nucleosomes in wild-type and *Nurf301* mutant hemocytes at all BEAF sites ordered according to BEAF signal. CP190 signal is shown for comparison. (B) Heatmap of nucleosomes in wild-type and *Nurf301* mutant hemocytes at BEAF sites that contain CP190 ordered according to CP190 signal. CP190 and BEAF signals are shown for comparison. (C) Heatmap of nucleosomes in wild-type and *Nurf301* mutant hemocytes at BEAF sites that lack CP190 ordered according to BEAF signal. BEAF signal is shown for comparison. Graph shows averaged nucleosome probability at BEAF sites that either contain (+CP190) or lack (-CP190) in wild-type and *Nurf301* mutant backgrounds.
(TIF)

S12 Fig Nucleosome reorganization at Mod(mdg4) sites. (A) Heatmap of nucleosomes in wild-type and *Nurf301* mutant hemocytes at Mod(mdg4)2.2 sites that contain CP190 ordered according to Mod(mdg4)2.2 signal. Mod(mdg4)2.2 and CP190 signals are shown for comparison. (B) Heatmap of nucleosomes in wild-type and *Nurf301* mutant hemocytes at Mod(mdg4) sites that contain CP190 ordered according to Mod(mdg4) signal. CP190 and Mod(mdg4) signal is shown for comparison. (C) Heatmap of nucleosomes in wild-type and *Nurf301* mutant hemocytes at Mod(mdg4) sites that lack CP190 ordered according to Mod(mdg4) signal. Graphs show averaged nucleosome probability at Mod(mdg4)2.2 and Mod(mdg4) sites that either contain (+CP190) or lack (-CP190) in wild-type and *Nurf301* mutant backgrounds.
(TIF)

S13 Fig Nucleosome reorganization at CP190 sites. (A) Heatmap of nucleosomes in wild-type and *Nurf301* mutant hemocytes at CP190 sites ordered according to CP190 signal. (B) CTCF, Su(Hw), Mod(mdg4) signals are shown for comparison. (C) BEAF and Mod(mdg4)2.2 signal is shown for comparison. Graph shows averaged nucleosome probability at CP190 sites in wild-type and *Nurf301* mutant backgrounds.
(TIF)

S1 Table Primer sequences for real-time PCR analysis. List of primers used in RT-PCR analysis of gene expression levels.
(DOCX)
