# Supplementary figures and images for: Genome-Wide Mapping Targets of the Metazoan Chromatin Remodeling Factor NURF Reveals Nucleosome Remodeling at Enhancers, Core Promoters and Gene Insulators
Source: PLoS Genet. 2016 Apr 5;12(4):e1005969. doi: 10.1371/journal.pgen.1005969 (PMC4821604; doi:10.1371/journal.pgen.1005969)

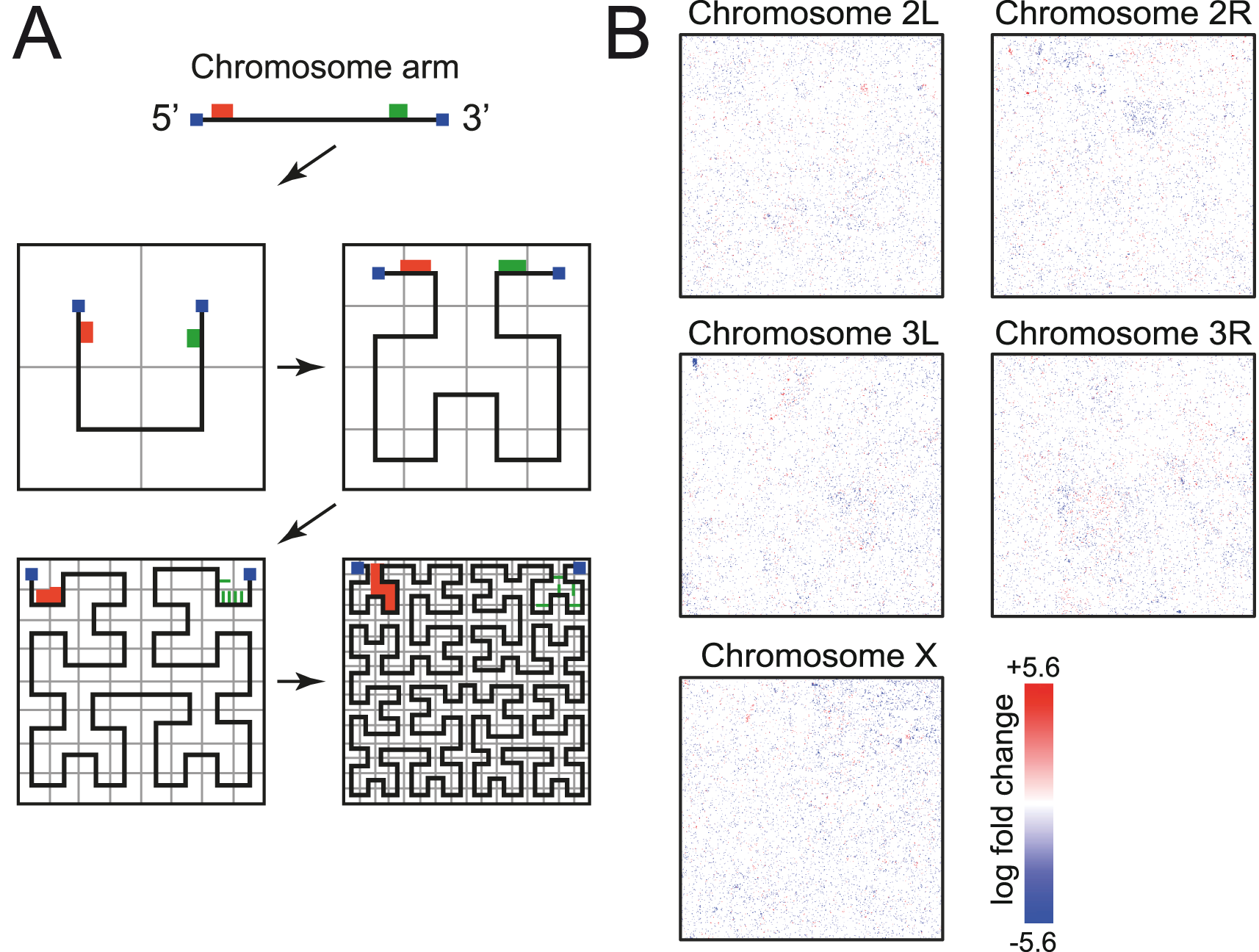

Supplement: S1 Fig — (A) Schematic illustrating a Hilbert plot. These are continuous space-filling curves in which one-dimensional data (in this case location of nucleosome shifts along a chromosome arm) is mapped in two dimensions allowing visualization of all data simultaneously. In this procedure a unit square is progressively divided into smaller quadrants and the one-dimensional line corresponding to each chromosome arm folded such that it passes through the center of each quadrant. The iterative folding has the advantage of allowing all data to points to be observed simultaneously and preserves locality, points that co-localize on the line typically co-map on the curve. In the example two features are plotted—in red (closely juxtaposed arrays of peaks), and in green peaks that are well separated from their neighbours. When the entire chromosome arm is viewed as in a genome browser window, it is impossible to discriminate between these two features due to the low resolution of the whole chromosome arm view. However, if these features are plotted as a Hilbert plot, as the linear arm is folded through smaller and smaller quadrants resolution increases and it becomes possible to discriminate closely juxtaposed and well separated features. In this context, extended domains of nucleosome re-organization would result in clusters of shifts being detected as extended clusters/domains on the Hilbert plot. (B) Hilbert plots of NURF-dependent nucleosome shifts on all chromosome arms. (TIF) [file pgen.1005969.s001.tif]

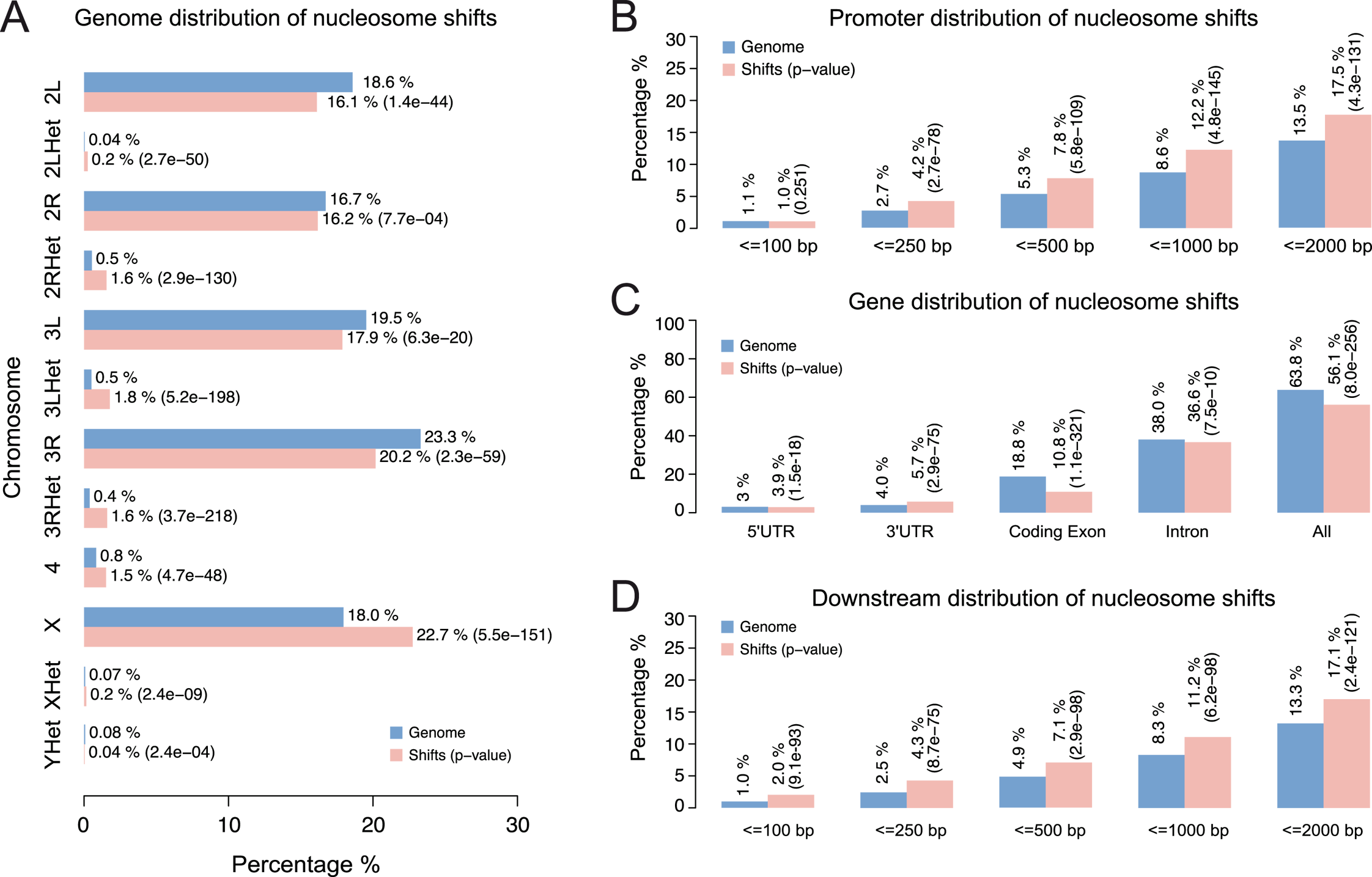

Supplement: S2 Fig — (A) Distribution of nucleosome shifts relative to chromosome arms compared with genome average of features. (B) Promoter distribution of nucleosome shifts compared with genome average of features. (C) Gene body distribution of nucleosome shifts compared with genome average of features. (D) Distribution of nucleosome shifts downstream of terminators compared with genome average of features. (TIF) [file pgen.1005969.s002.tif]

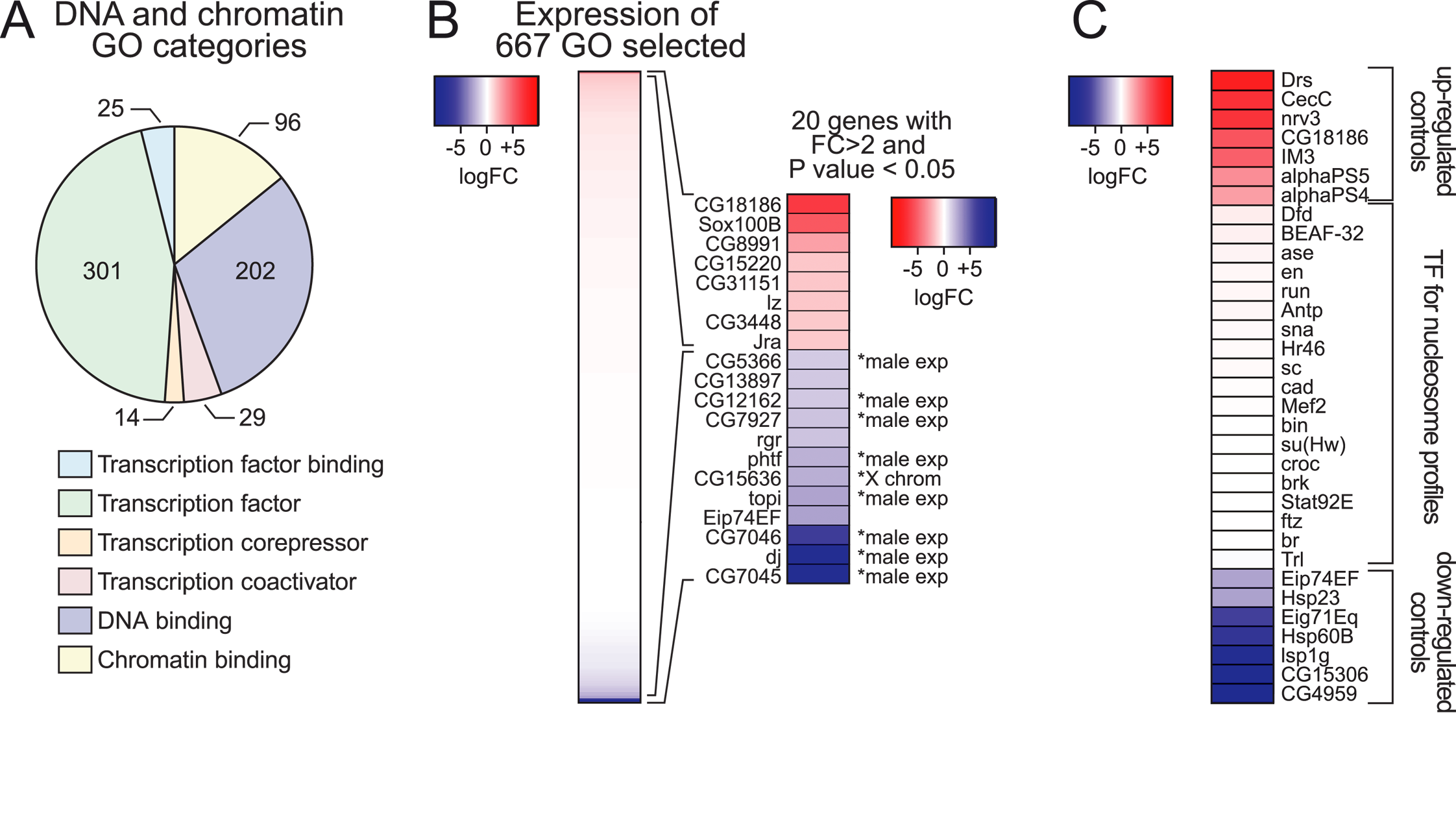

Supplement: S3 Fig — (A) To distinguish whether Nurf301 mutation affects expression of transcription factors or chromatin regulators that could indirectly affect nucleosome organisation in Nurf301 mutant hemocytes GO Molecular Function assignments (Flybase) were used to identify 667 targets of interest. Venn diagram indicates respective sub-categories of molecular function. (B) Expression of this set of 667 genes was analysed in Nurf301 mutant hemocytes relative to control wild-type hemocytes. Eight genes display elevated expression in Nurf301 mutant hemocytes, twelve show reduced expression. Of these, nine are male-expressed genes and, as nucleosome profiles were analysed in female hemocytes, these could be excluded from the analysis. None of the remaining eleven genes have known functions in control of global chromatin organisation or nucleosome positioning suggesting that the effects of Nurf301 mutants on nucleosome organisation are unlikely to be mediated through indirect effects on expression of other factors. (C) No change in expression of transcription factors previously analysed for effects on nucleosome organisation was observed. Expression of selected genes previously identified to be up-regulated (up-regulated controls) or down-regulated (down-regulated controls) in Nurf301 mutant hemocytes are displayed as controls. Change in expression is listed as log fold change in Nurf301 mutant hemocytes relative to wild-type hemocytes. (TIF) [file pgen.1005969.s003.tif]

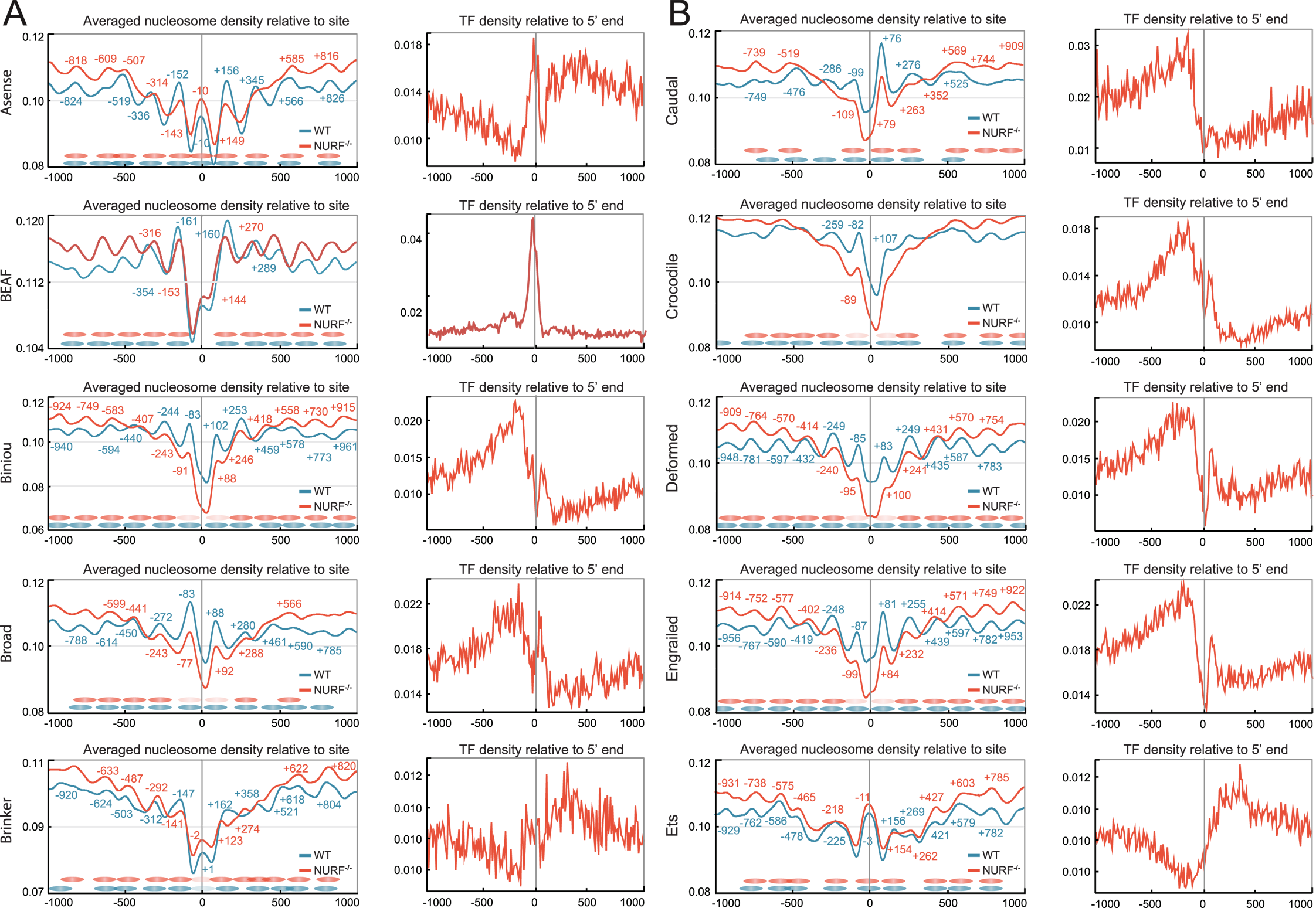

Supplement: S4 Fig — (A) Predicted binding sites for Drosophila TFs Asense, BEAF, Biniou, Broad, Brinker, Caudal, Crocodile, Deformed, Engrailed, Ets were determined using MEME and averaged nucleosome probability plots flanking predicted TF sites generated for both wild type (WT) and Nurf301 mutant populations. Five categories of nucleosome organization around TF sites could be distinguished. Dyad position of the +1 nucleosome is labeled. (B) Averaged profile plots of predicted TF-binding sites relative to the TSS. (TIF) [file pgen.1005969.s004.tif]

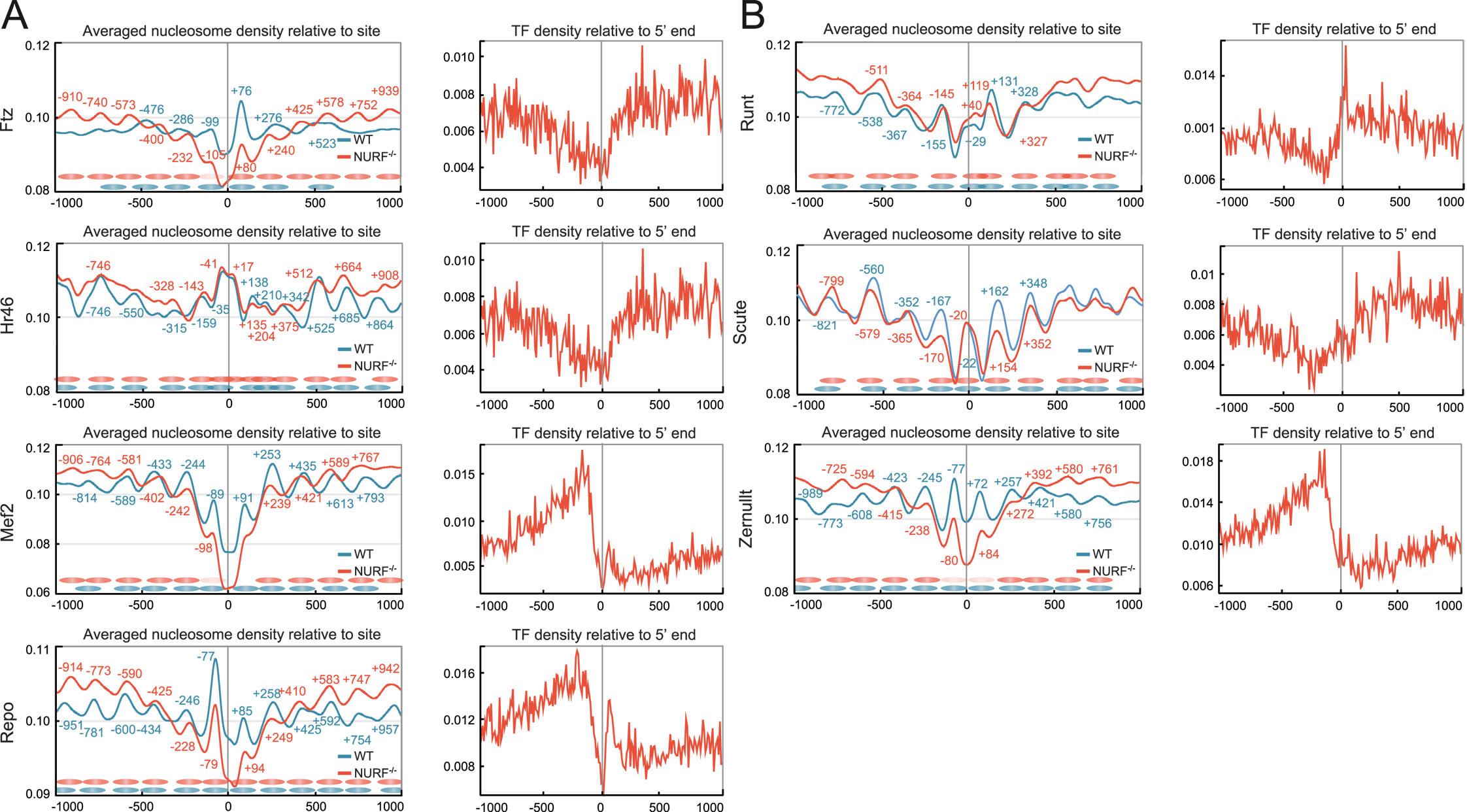

Supplement: S5 Fig — (A) Predicted binding sites for Drosophila TFs Ftz, Hr46, Mef2, Repo, Runt, Scute and Zerknult were determined using MEME and averaged nucleosome probability plots flanking predicted TF sites generated for both wild type (WT) and Nurf301 mutant populations. Five categories of nucleosome organization around TF sites could be distinguished. Dyad position of the +1 nucleosome is labeled. (B) Averaged profile plots of predicted TF-binding sites relative to the TSS. (TIF) [file pgen.1005969.s005.tif]

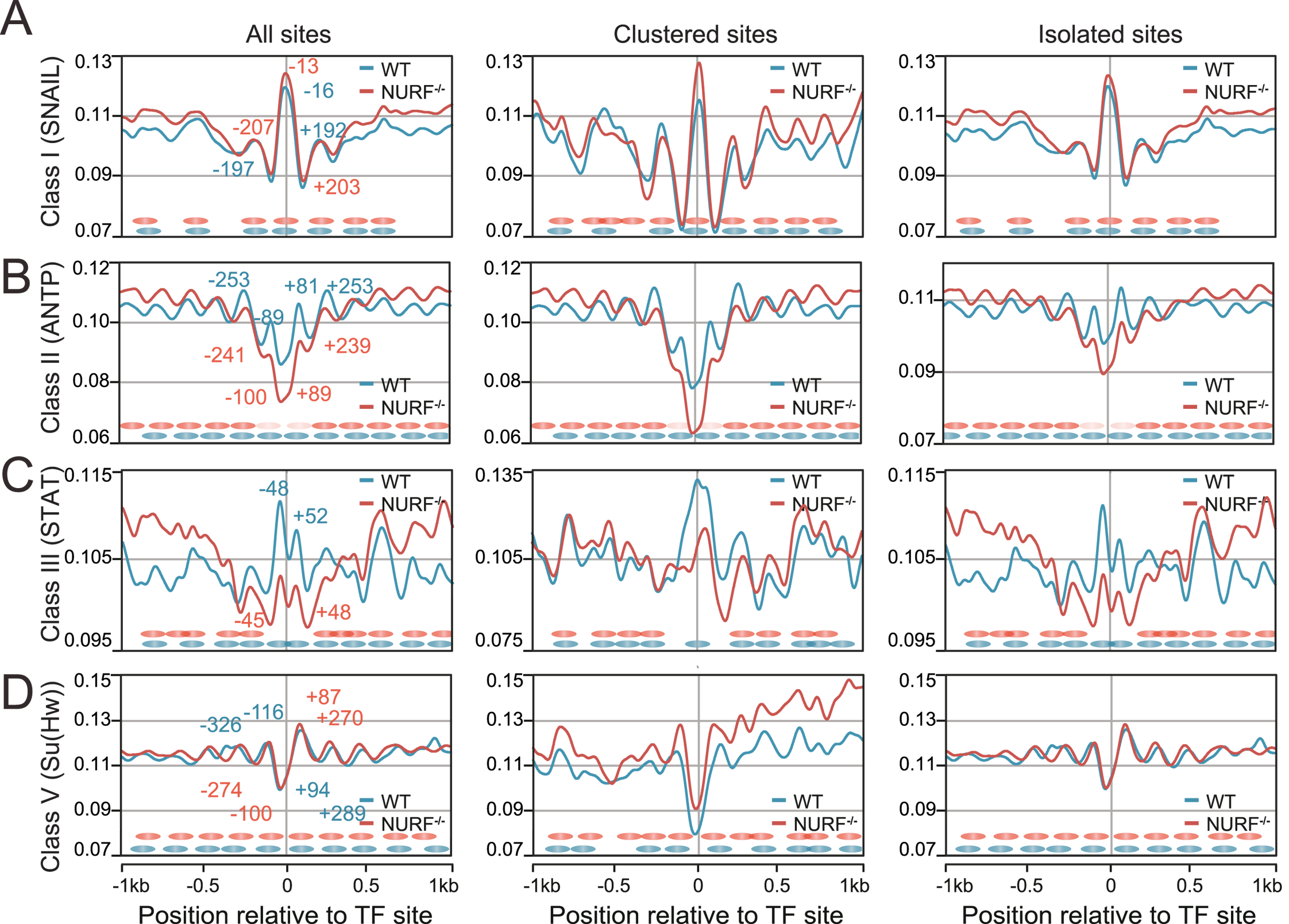

Supplement: S6 Fig — To discriminate whether nucleosome profiles were affected by clustering of TF binding sites, sites for (A) Snail, (B) Antp, (C) Stat and (D) Su(Hw) were divided into those with at least 2 sites within 50 bp (clustered sites) or those that did not possess neighbouring sites (isolated). Averaged nucleosome probability plots flanking these sites generated for both wild type (WT) and Nurf301/E(bx) mutants. (TIF) [file pgen.1005969.s006.tif]

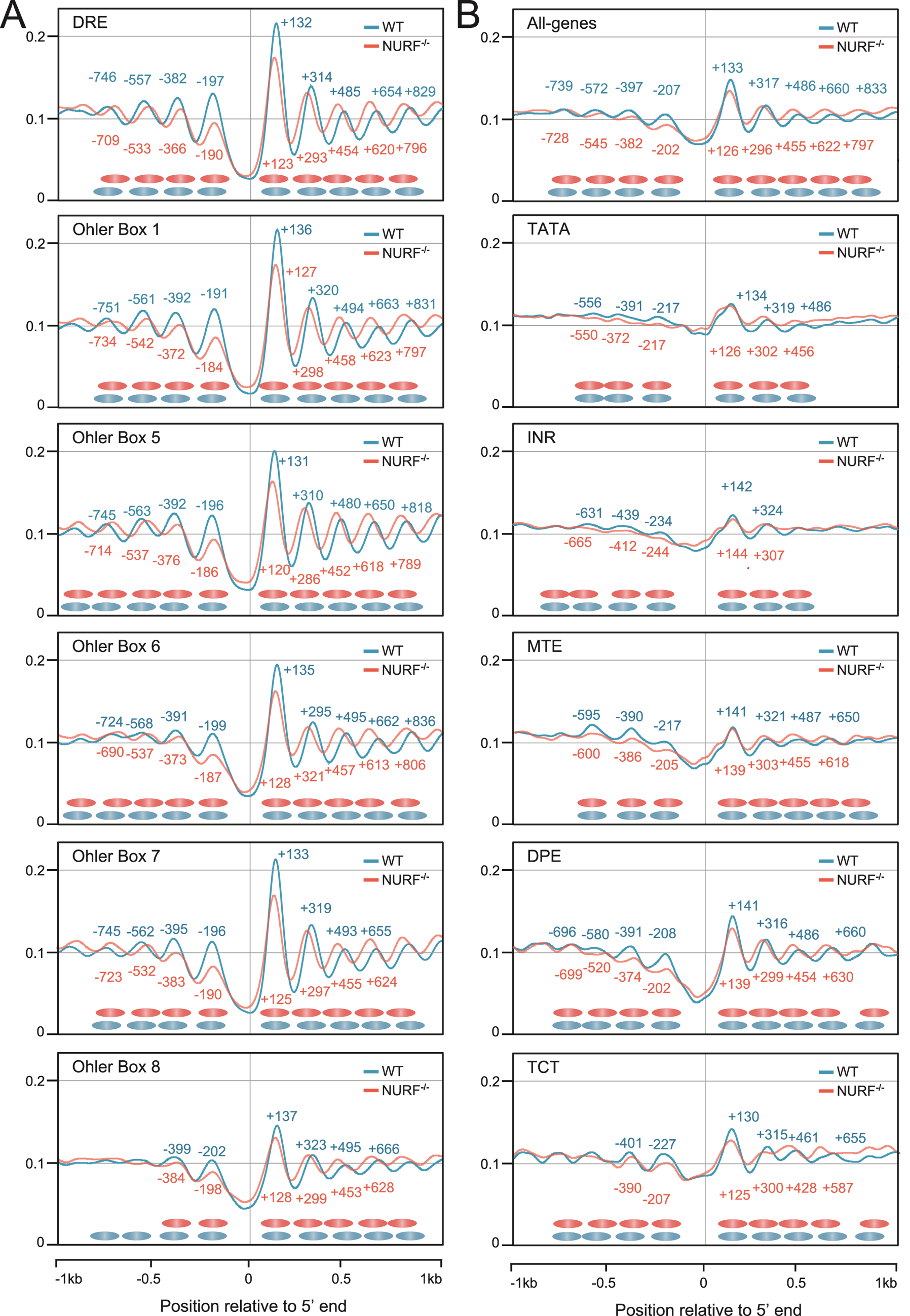

Supplement: S7 Fig — (A) TSSs were categorized based on core promoter motifs associated with developmentally regulated transcripts, TATA-box, initiator (INR) element, TCT motif, Motif Ten Element (MTE) and the downstream core promoter element (DPE). Averaged nucleosome probability plots flanking the TSS of each category generated for both wild type (WT) and Nurf301 mutants. (B) TSSs were categorized based on core promoter motifs associated with DREF/TRF2 targets, the DRE and Ohler Boxes 1,5,6,7,8. Averaged nucleosome probability plots flanking the TSS of each category generated for both wild type (WT) and Nurf301 mutants. Dyad position of the +1 nucleosome is labeled. (TIF) [file pgen.1005969.s007.tif]

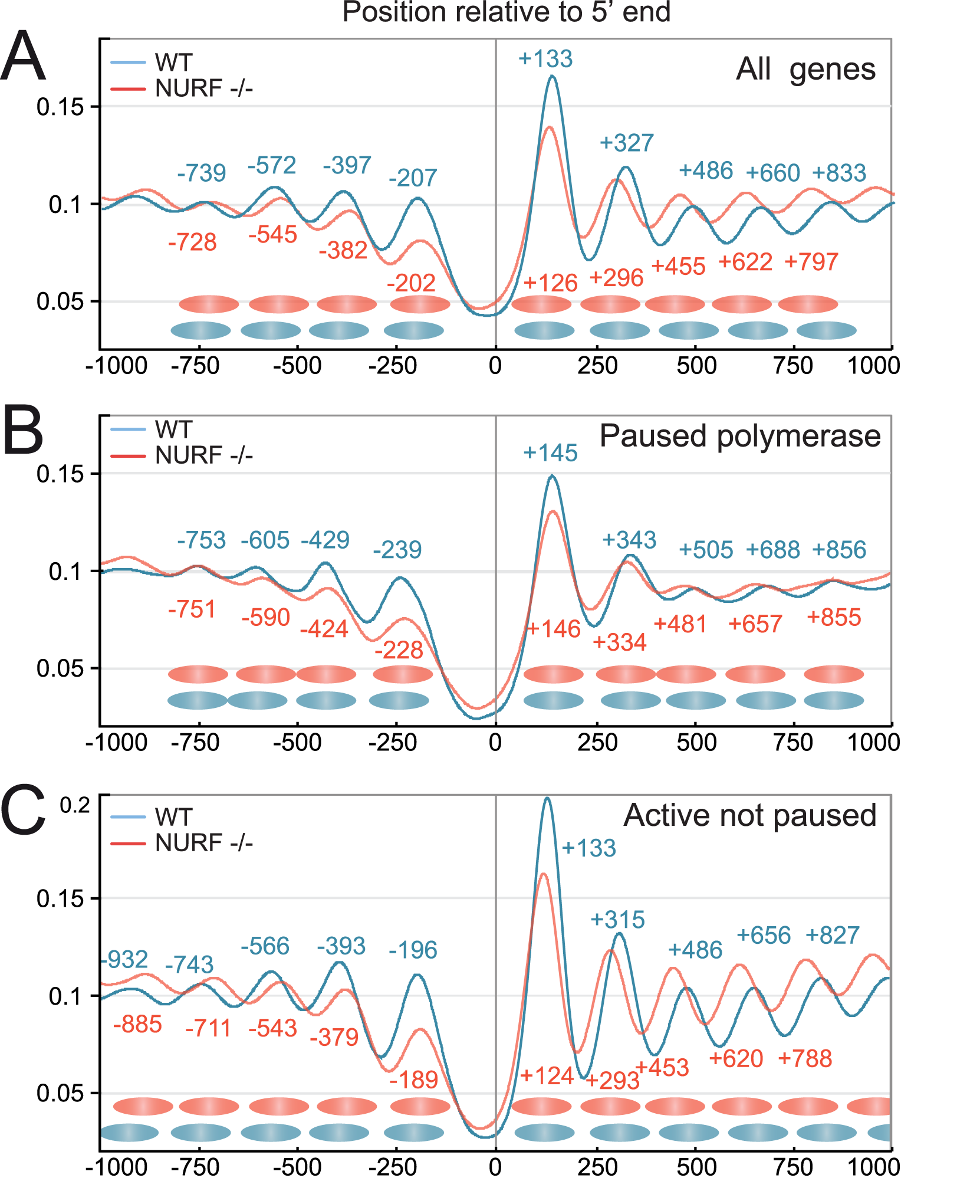

Supplement: S8 Fig — TSSs were categorized as stalled or active (not stalled). Averaged nucleosome probability plots flanking the TSS were generated for both wild type (WT) and Nurf301 mutants at (A) all TSSs, and TSSs with (B) paused polymerase or (C) active not stalled polymerase. Dyad position of the +1 nucleosome is labeled. (TIF) [file pgen.1005969.s008.tif]

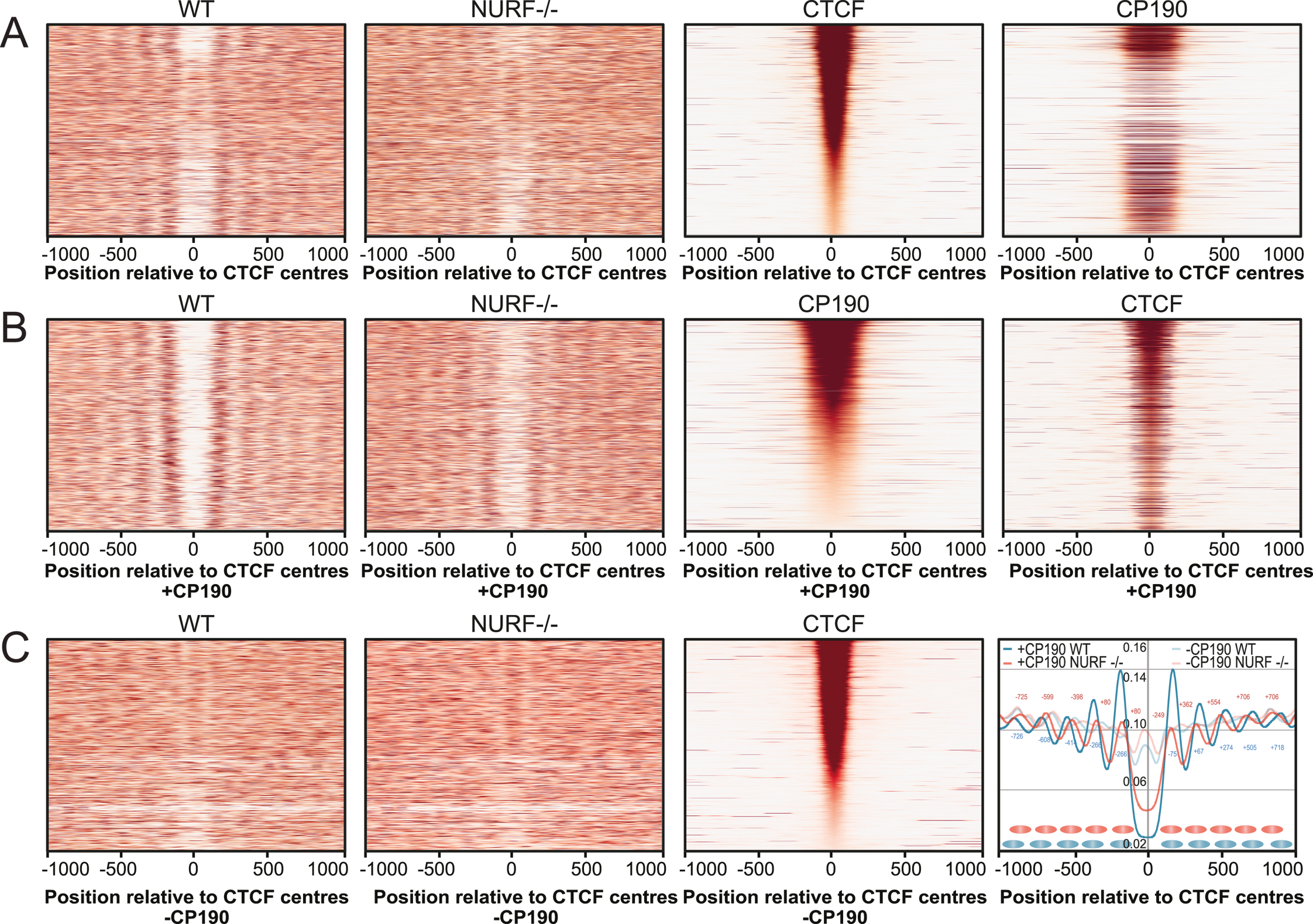

Supplement: S9 Fig — (A) Heatmap of nucleosomes in wild-type and Nurf301 mutant hemocytes at all CTCF sites ordered according to CTCF signal. CP190 signal is shown for comparison. (B) Heatmap of nucleosomes in wild-type and Nurf301 mutant hemocytes at CTCF sites that contain CP190 ordered according to CP190 signal. CP190 and CTCF signal is shown for comparison. (C) Heatmap of nucleosomes in wild-type and Nurf301 mutant hemocytes at CTCF sites that lack CP190 ordered according to CTCF signal. CTCF signal is shown for comparison. Graph shows averaged nucleosome probability at CTCF sites that either contain (+CP190) or lack (-CP190) in wild-type and Nurf301 mutant backgrounds. (TIF) [file pgen.1005969.s009.tif]

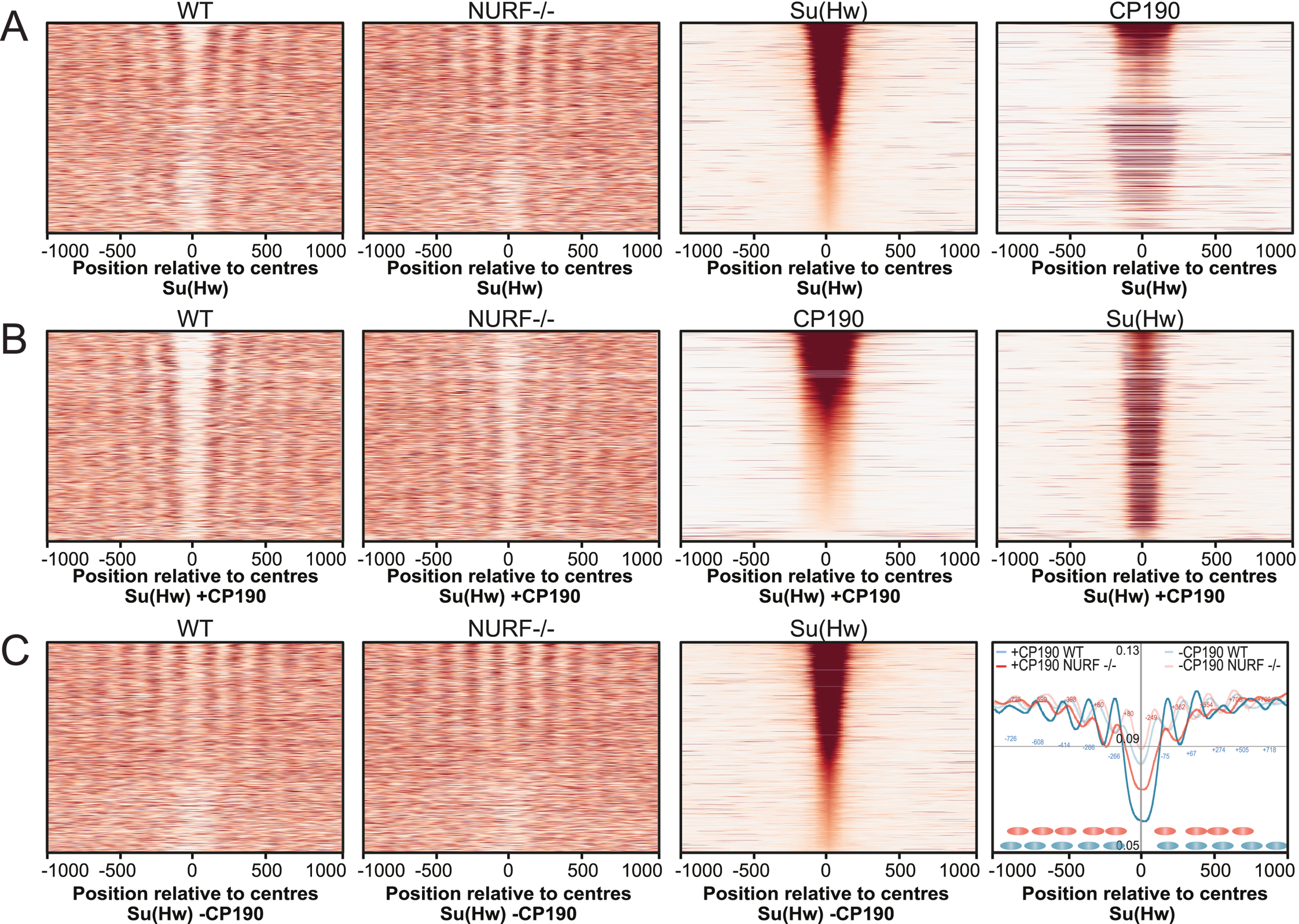

Supplement: S10 Fig — (A) Heatmap of nucleosomes in wild-type and Nurf301 mutant hemocytes at all Su(Hw) sites ordered according to Su(Hw) signal. CP190 signal is shown for comparison. (B) Heatmap of nucleosomes in wild-type and Nurf301 mutant hemocytes at Su(Hw) sites that contain CP190 ordered according to CP190 signal. CP190 and Su(Hw) signal is shown for comparison. (C) Heatmap of nucleosomes in wild-type and Nurf301 mutant hemocytes at Su(Hw) sites that lack CP190 ordered according to Su(Hw) signal. Su(Hw) signal is shown for comparison. Graph shows averaged nucleosome probability at Su(Hw) sites that either contain (+CP190) or lack (-CP190) in wild-type and Nurf301 mutant backgrounds. (TIF) [file pgen.1005969.s010.tif]

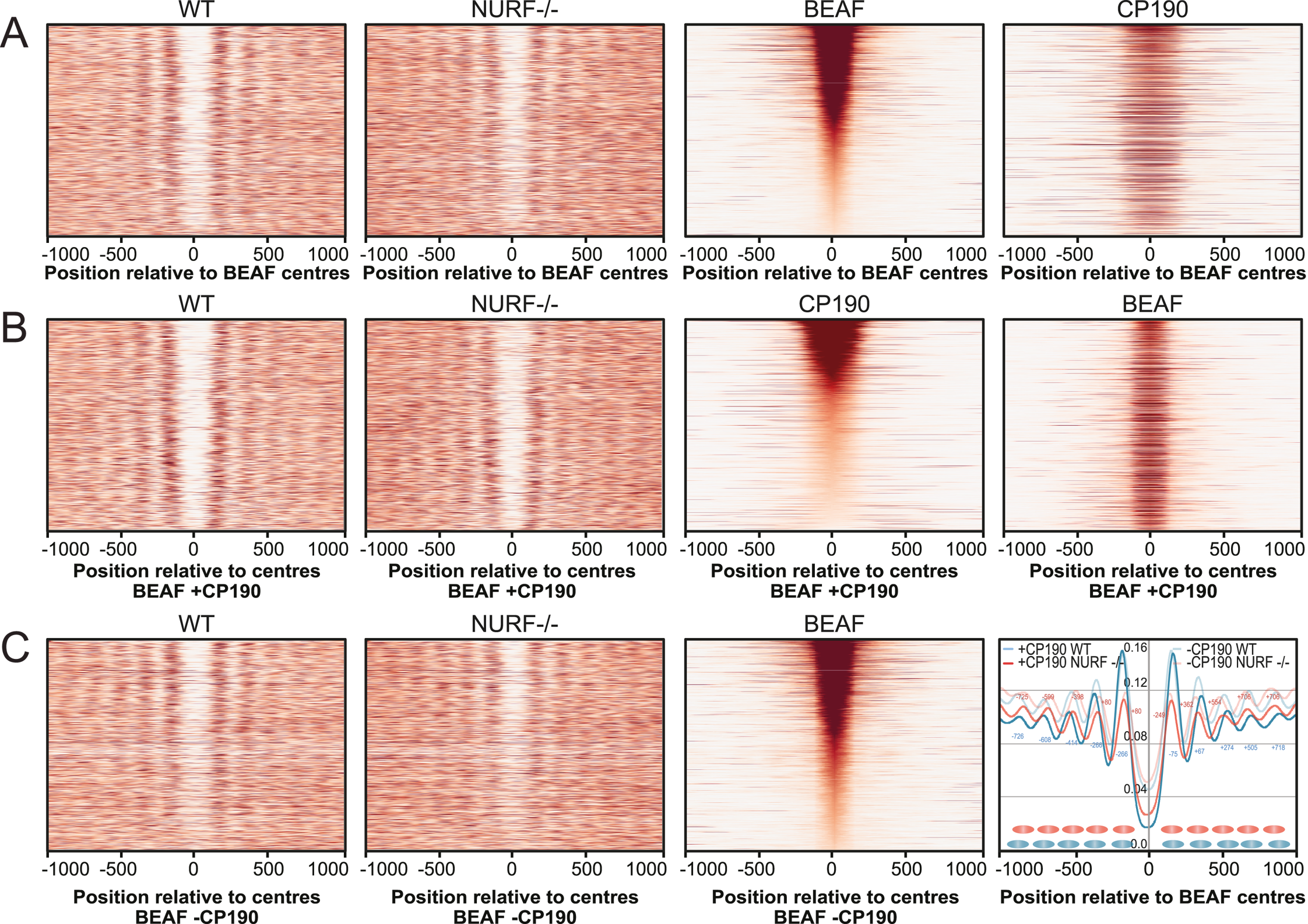

Supplement: S11 Fig — (A) Heatmap of nucleosomes in wild-type and Nurf301 mutant hemocytes at all BEAF sites ordered according to BEAF signal. CP190 signal is shown for comparison. (B) Heatmap of nucleosomes in wild-type and Nurf301 mutant hemocytes at BEAF sites that contain CP190 ordered according to CP190 signal. CP190 and BEAF signals are shown for comparison. (C) Heatmap of nucleosomes in wild-type and Nurf301 mutant hemocytes at BEAF sites that lack CP190 ordered according to BEAF signal. BEAF signal is shown for comparison. Graph shows averaged nucleosome probability at BEAF sites that either contain (+CP190) or lack (-CP190) in wild-type and Nurf301 mutant backgrounds. (TIF) [file pgen.1005969.s011.tif]

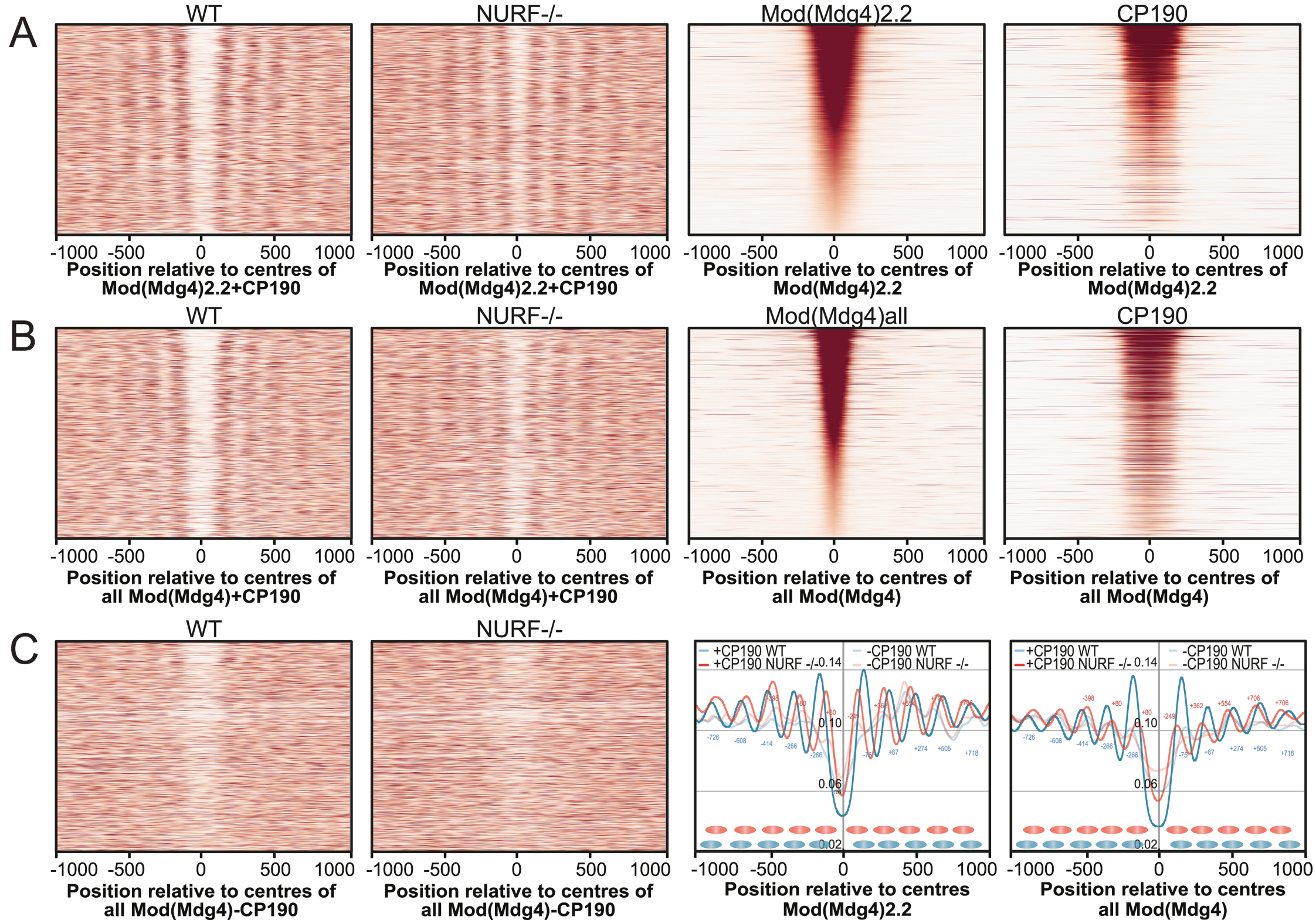

Supplement: S12 Fig — (A) Heatmap of nucleosomes in wild-type and Nurf301 mutant hemocytes at Mod(mdg4)2.2 sites that contain CP190 ordered according to Mod(mdg4)2.2 signal. Mod(mdg4)2.2 and CP190 signals are shown for comparison. (B) Heatmap of nucleosomes in wild-type and Nurf301 mutant hemocytes at Mod(mdg4) sites that contain CP190 ordered according to Mod(mdg4) signal. CP190 and Mod(mdg4) signal is shown for comparison. (C) Heatmap of nucleosomes in wild-type and Nurf301 mutant hemocytes at Mod(mdg4) sites that lack CP190 ordered according to Mod(mdg4) signal. Graphs show averaged nucleosome probability at Mod(mdg4)2.2 and Mod(mdg4) sites that either contain (+CP190) or lack (-CP190) in wild-type and Nurf301 mutant backgrounds. (TIF) [file pgen.1005969.s012.tif]

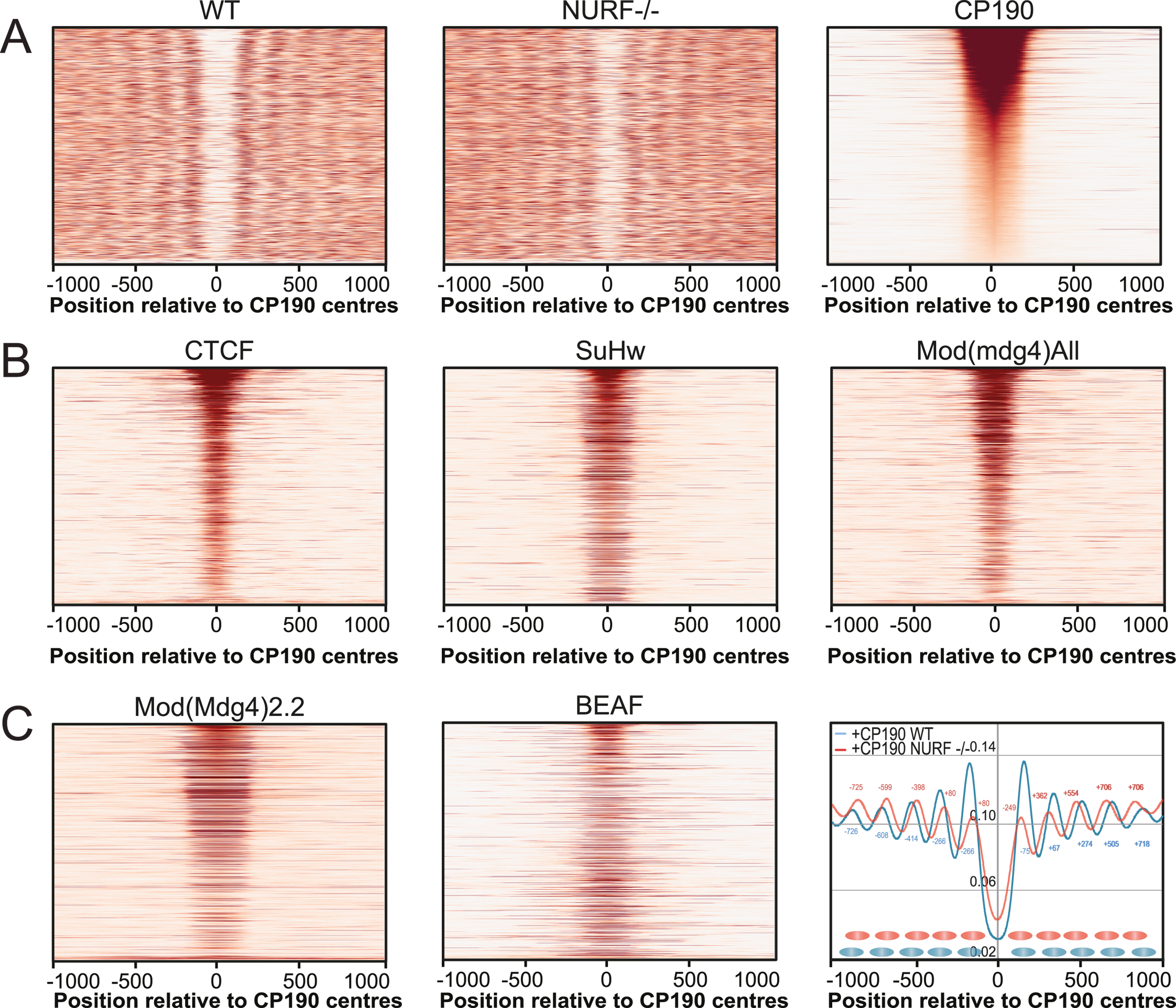

Supplement: S13 Fig — (A) Heatmap of nucleosomes in wild-type and Nurf301 mutant hemocytes at CP190 sites ordered according to CP190 signal. (B) CTCF, Su(Hw), Mod(mdg4) signals are shown for comparison. (C) BEAF and Mod(mdg4)2.2 signal is shown for comparison. Graph shows averaged nucleosome probability at CP190 sites in wild-type and Nurf301 mutant backgrounds. (TIF) [file pgen.1005969.s013.tif]
