# Supplementary material for: Genome-Wide Mapping Targets of the Metazoan Chromatin Remodeling Factor NURF Reveals Nucleosome Remodeling at Enhancers, Core Promoters and Gene Insulators
Source: PLoS Genet. 2016 Apr 5;12(4):e1005969. doi: 10.1371/journal.pgen.1005969 (PMC4821604; doi:10.1371/journal.pgen.1005969)
Supplement: S1 Table — List of primers used in RT-PCR analysis of gene expression levels. (DOCX) [file pgen.1005969.s014.docx]

**S1 Table: Primer sequences for real-time PCR analysis.**

| **Gene** | **Name of Primer** | **Primer Sequence (5’-3’)** |
| --- | --- | --- |
| *CG12220* | *CG12220_F* | CTTAGAGAGCTTGTTCCTGC |
|  | *CG12220_R* | CGTCCCCAATAAGCTGCTTC |
| *CG14512* | *CG14512_F* | CTCAAAGCTCTACAGAACCG |
|  | *CG14512_R* | GCTCTATCTGGACGTAG |
| *CG10681* | *CG10681_F* | CTTGCGGATGTGCACCAGTC |
|  | *CG10681_R* | CCTGATTCACCATTCCTGCG |
| *CG31111* | *CG31111_F* | CGGATGAGATCTGTCGTCTG |
|  | *CG31111_R* | CACTGGGTCGTCCACTGTTG |
| *CG31109* | *CG31109_F* | CAGCTGAGGATATAGCCAAC |
|  | *CG31109_R* | CGGTGCATCTCTGAACACAG |
| *CG15525* | *CG15525_F* | CATGATGGACAACAATGGCG |
|  | *CG15525_R* | CGGCAGCGTCTCCTCCTTTG |
| *CG11820* | *CG11820_F* | CGAGAACTACGATGACGATG |
|  | *CG11820_R* | CCGATCCGCTCCTTGATGCG |
| *CG10669* | *CG10669_F* | CGTCGAACACACGAGCTGTG |
|  | *CG10669_R* | CTCAAACAGGTGGATGGGTG |
| *CG4730* | *CG4730_F* | CCAAATGCTGGACATGACCG |
|  | *CG4730_R* | GTTTGCACAAGGTTCGTGGC |
| *CG5039* | *CG5039_F* | CCTCAGACGACCATTAGTCC |
|  | *CG5039_R* | CATGGCGTAGACCAGTGCAG |
| *CG4743* | *CG4743_F* | GGAATGGTAGTGGACATAGC |
|  | *CG4743_R* | CAGGAGCGAGTCCTTTGTAG |
| *alphaPS4* | *alphaPS4_F* | ACACCGACTCCTTGACCATC |
|  | *alphaPS4_R* | TGAGCACGTTGGTTAGCTTG |
| *alphaPS5* | *alphaPS5_F* | ACTTCGGTTACTCCGTGGTG |
|  | *alphaPS5_R* | GCACCCACGTCATAGGAATC |
| *Mys* | *Mys_F* | GATCACGGTACATGCGAGTG |
|  | *Mys_R* | GTACCATGACCGGAGCAGAT |
| *rp49* | *rp49_F* | CAGCTCGCGCACGTTGTGCACCAGGAACTT |
|  | *rp49_R* | CAACAGAGTCGGTCGCCGCTTCAAGGGACA |
| *Hemese* | *Hemese_F* | GGGGGATCCTGTAAACTTAGTGTGAACTGCTCCGCC |
|  | *Hemese_R* | CCCGGGCTCGAGGTGCTCGGATAACGCACACTGCGT |
| *Cher240* | *Cher240_F* | CGGATCAGTACGAGGAGAAC |
|  | *Cher240_R* | GATCGATGGTCTTCAGGTGC |
| *Lsp1gamma* | *Lsp1gamma_F* | AGGAGCGCCTGGCCAACGGT |
|  | *Lsp1gamma_R* | GGACCTTGTAGACGCGACTG |

**Supplemental References**

Badenhorst P, Voas M, Rebay I, Wu C. 2002. Biological functions of the ISWI chromatin remodeling complex NURF. *Genes & development* **16**: 3186-3198.

Bai X, Larschan E, Kwon SY, Badenhorst P, Kuroda MI. 2007. Regional Control of Chromatin Organization by Noncoding roX RNAs and the NURF Remodeling Complex in Drosophila melanogaster. *Genetics* **176**: 1491-1499.

Blair SS. 2000. Imaginal discs. . in *Drosophila Protocols* (eds. W Sullivan, M Ashburner, RS Hawley), pp. 159-173,. Cold Spring Harbor Laboratory Press., Cold Spring Harbor.

Boyle AP, Guinney J, Crawford GE, Furey TS. 2008. F-Seq: a feature density estimator for high-throughput sequence tags. *Bioinformatics* **24**: 2537-2538.

Butcher RD, Chodagam S, Basto R, Wakefield JG, Henderson DS, Raff JW, Whitfield WG. 2004. The Drosophila centrosome-associated protein CP190 is essential for viability but not for cell division. *Journal of cell science* **117**: 1191-1199.

Giardine B, Riemer C, Hardison RC, Burhans R, Elnitski L, Shah P, Zhang Y, Blankenberg D, Albert I, Taylor J et al. 2005. Galaxy: a platform for interactive large-scale genome analysis. *Genome research* **15**: 1451-1455.

Kwon SY, Xiao H, Glover BP, Tjian R, Wu C, Badenhorst P. 2008. The nucleosome remodeling factor (NURF) regulates genes involved in Drosophila innate immunity. *Developmental biology* **316**: 538-547.

Kwon SY, Xiao H, Wu C, Badenhorst P. 2009. Alternative splicing of NURF301 generates distinct NURF chromatin remodeling complexes with altered modified histone binding specificities. *PLoS genetics* **5**: e1000574.

Langmead B, Trapnell C, Pop M, Salzberg SL. 2009. Ultrafast and memory-efficient alignment of short DNA sequences to the human genome. *Genome biology* **10**: R25.

Li H, Handsaker B, Wysoker A, Fennell T, Ruan J, Homer N, Marth G, Abecasis G, Durbin R. 2009. The Sequence Alignment/Map format and SAMtools. *Bioinformatics* **25**: 2078-2079.

Liu T, Ortiz JA, Taing L, Meyer CA, Lee B, Zhang Y, Shin H, Wong SS, Ma J, Lei Y et al. 2011. Cistrome: an integrative platform for transcriptional regulation studies. *Genome biology* **12**: R83.

Muse GW, Gilchrist DA, Nechaev S, Shah R, Parker JS, Grissom SF, Zeitlinger J, Adelman K. 2007. RNA polymerase is poised for activation across the genome. *Nat Genet* **39**: 1507-1511.

Oegema K, Whitfield WG, Alberts B. 1995. The cell cycle-dependent localization of the CP190 centrosomal protein is determined by the coordinate action of two separable domains. *The Journal of cell biology* **131**: 1261-1273.

Van Bortle K, Ramos E, Takenaka N, Yang J, Wahi JE, Corces VG. 2012. Drosophila CTCF tandemly aligns with other insulator proteins at the borders of H3K27me3 domains. *Genome research* **22**: 2176-2187.

Wu Z, Irizarry RA, Gentleman R, Martinez-Murillo F, Spencer F. 2004. A Model-Based Background Adjustment for Oligonucleotide Expression Arrays. *Journal of the American Statistical Association* **99**: 909-917.

Wysocka J, Reilly PT, Herr W. 2001. Loss of HCF-1-chromatin association precedes temperature-induced growth arrest of tsBN67 cells. *Molecular and cellular biology* **21**: 3820-3829.

Wysocka J, Swigut T, Xiao H, Milne TA, Kwon SY, Landry J, Kauer M, Tackett AJ, Chait BT, Badenhorst P et al. 2006. A PHD finger of NURF couples histone H3 lysine 4 trimethylation with chromatin remodelling. *Nature* **442**: 86-90.

Zhang Y, Liu T, Meyer C, Eeckhoute J, Johnson D, Bernstein B, Nusbaum C, Myers R, Brown M, Li W et al. 2008. Model-based Analysis of ChIP-Seq (MACS). *Genome biology* **9**: R137.
